# Global incidence and risk factors for glaucoma: A systematic review and meta-analysis of prospective studies

**DOI:** 10.7189/jogh.14.04252

**Published:** 2024-11-08

**Authors:** Shiyi Shan, Jing Wu, Jin Cao, Yan Feng, Jiali Zhou, Zeyu Luo, Peige Song, Igor Rudan, Global Health Epidemiology Research Group (GHERG)

**Affiliations:** 1 Centre for Clinical Big Data and Statistics of the Second Affiliated Hospital, Zhejiang University School of Medicine, School of Public Health, Zhejiang University School of Medicine, Hangzhou, China; 2 School of Oral Medicine, Zhejiang University School of Medicine, Zhejiang University, Hangzhou, China; 3 Centre for Global Health, Usher Institute, University of Edinburgh, Edinburgh, Scotland, UK

## Abstract

**Background:**

This study aims to estimate global incidence and assess risk factors for glaucoma subtypes.

**Methods:**

The literature search was performed in three English (PubMed, Embase, MEDLINE) and three Chinese (China National Knowledge Infrastructure, Wanfang, China Science and Technology Journal Database) databases to identify prospective studies on glaucoma incidence between 1 January 1990 and 29 November 2022. We used a multilevel mixed-effects meta-regression to estimate the age- and sex-specific incidence rate of primary open-angle glaucoma (POAG). The global and regional incidence rate of POAG in 2022 were respectively estimated. The annual cumulative incidence (ACI) of POAG and primary angle-closure glaucoma (PACG), and risk factors for POAG were pooled using a random-effects meta-analysis, respectively. The heterogeneity of the included articles was tested using the Q statistic and measured by *I^2^* index. Publication bias was detected by funnel plots, Egger's regression test, and Begg's rank correlation test.

**Results:**

A total of 9050 articles were identified in literature search, and 50 articles provided incidence data of glaucoma subtypes. In 2022, the global incidence rate of POAG was 23.46 (95% confidence interval (CI) = 15.68–32.91) per 10 000 person-years among 40–79 years. An increase from 5.51 (95% CI = 1.63–11.12) per 10 000 person-years in 40–44 years to 64.36 (95% CI = 49.82–80.70) per 10 000 person-years in 75–79 years was noted between the year 1990 and 2019. Across sociodemographic index (SDI) and World Health Organization (WHO) regions, the incidence rate was the highest in low SDI region and Africa, respectively. The pooled ACI of POAG was 0.21% (95% CI = 0.13%–0.30%). Six risk factors for POAG were identified, including intraocular pressure (IOP) treatment (meta-odds ratio (OR) = 3.69; 95% CI = 2.64–5.15), a family history of glaucoma (meta-OR = 2.49; 95% CI = 1.92–3.24), myopia (meta-OR = 2.08; 95% CI = 1.59–2.70), elevated IOP (meta-OR = 1.13; 95% CI = 1.11–1.15), advanced age (meta-OR = 1.07; 95% CI = 1.05–1.08), male (female: meta-OR = 0.76; 95% CI = 0.66–0.88). The pooled ACI of PACG was 0.05% (95% CI = 0.00%–0.16%).

**Conclusions:**

Significant disparities existed in incidence rates for glaucoma across geographic regions and age groups. Further research is needed to understand which risk factors drive glaucoma incidence in different socioeconomic strata for tailored health policy on preventing glaucoma.

**Registration:**

This study is registered with PROSPERO (number CRD42023434203).

Glaucoma, characterised by progressive optic neuropathy, is a leading cause of irreversible visual impairment and blindness worldwide [1]. In 2020, an estimated 76 million individuals globally were affected by glaucoma, with approximately 4.5 million experiencing moderate to severe visual impairment and 3.2 million suffering from blindness [1]. With the anticipated rise in the aging population, the number of prevalent glaucoma cases is projected to reach 112 million by 2040 [2]. Glaucoma-induced visual impairment seriously affects the life quality and healthy life expectancy of patients. It also imposes a large burden on families and health care systems [3–5]. Due to the insidious onset and low detection rate in the early stages of glaucoma, most patients are diagnosed in advanced stages with extensive and irreversible damage [6–8]. Therefore, early detection and intervention are essential to prevent irreversible visual damage and associated disability [9].

Glaucoma could be grouped into several subtypes, as classified by the tenth revision of the International Classification of Diseases, mainly including primary open-angle glaucoma (POAG), primary angle-closure glaucoma (PACG), secondary glaucoma, and congenital glaucoma. The incidence of glaucoma and its subtypes varies significantly across different ethnicities and geographies. For instance, the Singapore Epidemiology of Eye Diseases study reported that the six-year cumulative incidences of PACG were 0.13, 0.59, and 0.05% for Malay, Indian, and Chinese Singaporeans aged over 40 years, respectively [10]. Similarly, a cohort study focused on the Indian population in Singapore aged between 40 and 80 years found a six-year cumulative incidence of primary glaucoma, POAG, and PACG at 1.68, 1.37, and 0.32%, respectively [11]. Additionally, the Yunnan Minority Eye Study revealed a five-year cumulative incidence of POAG at 1.3% among the Bai ethnic group aged 55–95 years in China [12]. The heterogeneity of incidence rates, partly due to disparities in age distribution, sample size, geography, and ethnicity across studies, underscores the need for a systematic and comprehensive analysis of the global epidemiological status of glaucoma and its subtypes.

Risk factors for glaucoma are multifaceted, with some nonmodifiable such as a family history of the disease, which can increase risk by up to 2.85 times [13,14]. Other documented risk factors include advanced age, male sex, high intraocular pressure (IOP), and high blood pressure (BP) [15–17]. Moreover, glaucoma can also develop secondary to certain systemic diseases or treatments, such as diabetes, hypothyroidism, and corticoid hormone use [18–21]. All the aforementioned risk factors may individually or collectively influence the risk of glaucoma. The identification and management of modifiable risk factors are needed to reduce the incidence and burden of glaucoma.

To fill the research gap, this study conducted a comprehensive systematic review and meta-analysis to synthesise the available evidence since 1990, aiming to:

1) quantify the global incidence of glaucoma and its subtypes

2) identify risk factors for the development of glaucoma.

## METHODS

This systematic review was conducted and reported in line with the Preferred Reporting Items for Systematic Reviews and Meta-Analyses guidelines and the Guidelines for Accurate and Transparent Health Estimates Reporting statement [22,23]. This systematic review has been prospectively registered on PROSPERO (ID: CRD42023434203).

### Literature search

To identify articles reporting the incidence of glaucoma, a comprehensive literature search was performed across three English (PubMed, Embase, MEDLINE) and three Chinese bibliographic databases (China National Knowledge Infrastructure, Wanfang, China Science and Technology Journal Database). The search strategy combined terms related to glaucoma (e.g. ‘glaucoma’, ‘POAG’, ‘PACG’), incidence (e.g. ‘new-onset’, ‘incidence’, ‘morbidity’), and prospective study design (e.g. ‘cohort’, ‘prospective’, ‘follow-up’, ‘longitudinal’), in forms of medical subject headings and free-text terms. The search was restricted to articles published between 1 January 1990 and 29 November 2022, without any language or geographic restrictions. The specific search strategies employed for each electronic database are detailed in Table S1 in the **Online Supplementary Document**. The reference lists of included articles were further scrutinised to complement the database searches.

### Selection criteria

All records identified from the six electronic databases were merged, with duplicates removed. Two researchers (SS and YF) independently screened all records in two review stages: screening of titles and abstracts, followed by full-text examination.

The inclusion criteria were as follows:

1) prospective population-based study (community or health check-based) in the general population

2) articles that provided either cumulative incidence (or incidence proportion, the proportion of a given population that develops glaucoma during a specified time period) or incidence rate (or incidence density, accounting for the person-time at risk during the study period)

3) the incidence estimates were based on the number of individuals with glaucoma, rather than the number of eyes affected

4) articles with clear definition or examination method criteria for glaucoma.

The structural or functional indications of glaucomatous optic neuropathy are identified either by physician diagnosis or confirmation through optic disc examination and/or visual field assessment, rather than dependent IOP measurement.

Studies that were conducted in special populations with characteristics that clearly indicated it to be unrepresentative, e.g. in-patients/out-patients, or people with specific diseases (e.g. hypertension, diabetes, acquired immune deficiency syndrome) were excluded. Abstracts, case reports, reviews, viewpoints, and letters were also excluded. In instances of multiple publications from the same study, the one with the largest sample size was kept for inclusion. If the sample size were the same, the article with the most up-to-date data was kept.

### Data extraction and quality assessment

Data were independently extracted from the included articles by two researchers (SS and YF). This study focused on three major types of glaucoma, namely POAG, PACG, and secondary glaucoma. Relative data on different subtypes of glaucoma was extracted separately. The data extraction form included the following four modules:

1) bibliographic information: title, author(s), publication year, country, World Health Organization (WHO) region (Africa, Americas, Europe, Eastern Mediterranean, South-East Asia, and Western Pacific), study location, latitude, longitude, sociodemographic index (SDI) of the survey location at the median year of follow-up (high SDI, high-middle SDI, middle SDI, low-middle SDI, low SDI), study setting (urban, rural, or mixed), baseline date, follow-up period, sampling strategy, definition and diagnostic methods of glaucoma

2) characteristics of studies and participants: sample size, inclusion and exclusion criteria for the sample population, ethnicity, sex (male, female, or mixed), and age (age range, mean age, median age, or midpoint of the age range). In the case of censoring age band, the missing band was imputed by taking the same (or average) width reported in the same article

3) incidence estimates: for cumulative incidence – number of incident cases, sample size, and the duration of follow-up; for incidence rate – number of incident cases and aggregate person-years of observation. If incidence estimate within an article was stratified by age, sex, and setting, the corresponding stratified incidence rates were extracted

4) a subset of included prospective studies explored potential risk factors for glaucoma using multivariable regression, from which we extracted the corresponding definitions of risk factors, their hazard ratio (HR)/risk ratio (RR)/odds ratio (OR), and 95% confidence intervals (95% CIs).

The quality of the included articles was evaluated using the Newcastle-Ottawa Scale (NOS) [24]. The NOS quality assessment scale is composed of three modules (selection, comparability, outcome) and evaluates the quality of cohort studies based on eight items. According to the semiquantitative star system, each item in the comparability module could receive a maximum of two stars, while the remaining items could receive a maximum of one star. The maximum score was nine, with one star corresponding to one point. Articles were scored on a range of 0–9 points, and those with a score of five or above were considered to be of high quality [25] (Table S2 in the **Online Supplementary Document**).

Disagreements during the phases of article selection, data extraction, and quality assessment were resolved by consensus through discussion or by consultations with a senior researcher (PS). We assessed two researchers’ inter-rater reliability using percent agreement.

### Statistical analysis

#### Meta-regression of the incidence rate of POAG

Incidence rate refers to the number of new incident cases of glaucoma within the total observed person-years within a specific period as the denominator. Due to the limited number of articles that reported the incidence rate of PACG and secondary glaucoma, we only estimated the incidence rate of POAG. Some included articles reported incidence rates of POAG in groups stratified by age or sex. Cumulative incidence of POAG was transformed into incidence rate in articles with specific sample sizes and follow-up time, using the following formula:

Incidence rate = n / (N × t)

where is the number of incident cases, is the number of overall samples during the follow-up period, and is the time of follow-up.

Then, we combined these transformed data with extracted incidence rate of POAG to form the overall data set of POAG incidence rate. To investigate potential sources of heterogeneity between articles reporting the incidence rate of POAG, and to take this hierarchical data structure into consideration, a multilevel mixed-effect meta-regression was conducted. First, the associations of several cluster-level variables, including age, male-to-female ratio, WHO region (Africa, Americas, Europe, Eastern Mediterranean, South-East Asia, and Western Pacific), SDI, with POAG incidence rates were explored using a univariable regression (Table S3 in the **Online Supplementary Document**). Only age and male-to-female ratio were found to be factors that were significantly associated with POAG incidence rates. Thus, the age- and sex-specific incidence rates of POAG were estimated at the global level, as well as for different WHO regions and SDI groups. Given the degenerative nature of POAG, we assumed that the incidence rate of POAG before 30 years was zero. To enable the inclusion of zero cases as reported, we replaced zero cells with a value of 0.0005. For the estimation, we set the age range of interest to 40–79 years, where sufficient data were available for model construction.

#### Meta-analysis of the annual cumulative incidence of POAG and PACG

Cumulative incidence was defined as the number of new incident cases over the total number of individuals with glaucoma at a baseline during a specified period. As a rule, at least three articles were required for meta-analysis. Due to the limited number of included articles for secondary glaucoma, we only pooled the cumulative incidence of POAG and PACG. Since studies varied in the length of follow-up period, we calculated the annual cumulative incidence (ACI) as follows:

ACI = − ln (1 − S) / t

where is the proportion of new incident cases (number of new incident cases at follow-up divided by number at risk at baseline) over years, and is the time of follow-up [26].

With a random-effects meta-analysis, we pooled the ACI of POAG and PACG after Freeman-Tukey double arcsine transformation. The heterogeneity of the included articles was tested using the Q statistic and measured by *I^2^* index [27]. Q statistic with *P*-value <0.05 or *I^2^* index >50% indicated significant heterogeneity [28]. To examine whether single studies had a disproportionately excessive influence, a leave-one-out sensitivity analysis was conducted for each meta-analysis. The robustness of the meta-analysis results was evaluated by leave-one-out sensitivity analyses. Publication bias was detected by funnel plots, Egger's regression test, and Begg's rank correlation test. A trim-and-fill method was employed when publication bias was found.

#### Meta-analysis of risk factors for POAG

A subset of articles that reported the incidence of glaucoma also reported potential risk factors for glaucoma based on multivariable regression. We selected risk factors that have been reported in at least three individual articles. Finally, the effect size of eight risk factors for POAG – namely, age, sex, IOP, IOP treatment, family history of glaucoma, myopia, diabetes, and hypertension – were assessed with random-effects (restricted maximum-likelihood estimator method) meta-analyses.

We employed credit assessment to evaluate the validity of the associations between risk factors and POAG, and divided into groups based on the predetermined criteria [29,30] (Table S4 in the **Online Supplementary Document**). The credibility of evidence yielding significant results (*P* < 0.05) was divided into the following four categories: convincing (I), highly suggestive (II), suggestive (III), and weak (IV).

All statistical analyses were conducted using R version 4.2.1 (R Foundation for Statistical Computing, Vienna, Austria, 2022). A two-side *P*-value of less than 0.05 indicated statistical significance.

## RESULTS

### Summary of systematic review

**Figure 1:**
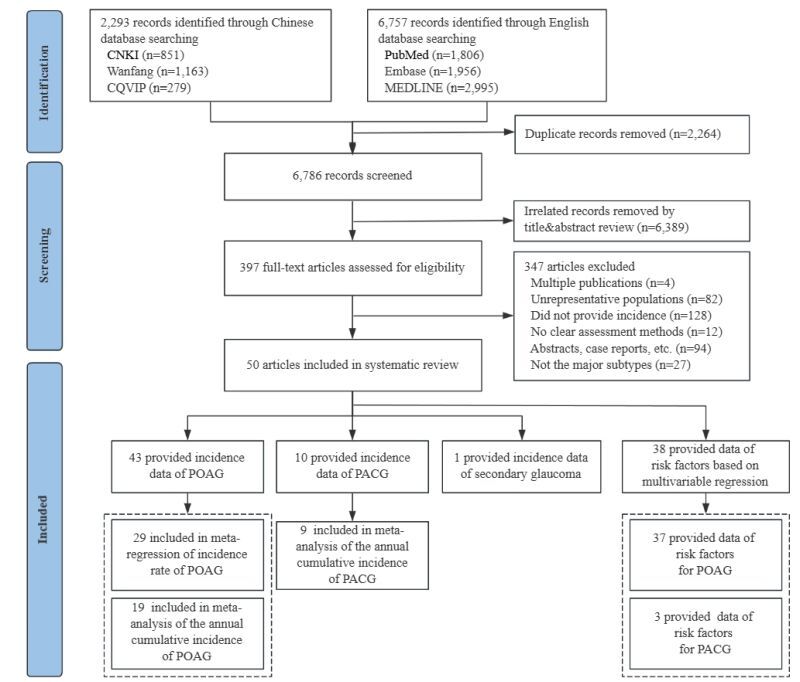
Flowchart of systematic review and meta-analysis. Four articles provided incidence data for both primary open-angle glaucoma and primary angle-closure glaucoma.

**Figure 2:**
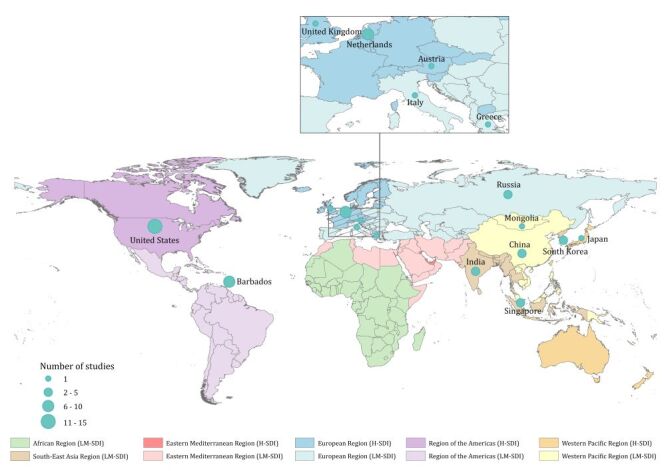
Location of included articles reporting the incidence or risk factors for glaucoma subtypes. H-SDI – high social demographic index countries, LM-SDI – low- and middle-sociodemographic index countries.

The process of systematic review is illustrated in **Figure 1**. A total of 9050 articles were initially identified in literature search. Following the removal of 2264 duplicates, the remaining 6786 articles underwent further screening. Throughout the title and abstract screening stage, as well as full-text screening stage, a total of 6389 and 347 articles were excluded, respectively. Ultimately, 50 articles provided incidence data of glaucoma subtypes. Inter-rater reliability for data extraction was 0.93 for included studies. The full list and detailed information of all included articles are shown in Table S5 and Table S6 in the **Online Supplementary Document**. Of the included articles, 43 articles (86.0%) reported the incidence of POAG, 10 articles (20.0%) reported the incidence of PACG, and one article (2.0%) reported the incidence of secondary glaucoma (with four articles simultaneously reporting the cumulative incidence of both POAG and PACG). Utilising multivariable regression, 38 articles reported on potential risk factors for POAG (37 articles, 97.4%) and PACG (three articles, 7.9%). Articles included in the systematic review did not cover the African and Eastern Mediterranean regions, with the majority focused on European Region and Region of the Americas (38 articles, 76.0%) (**Figure 2**). More than half (32 articles, 64.0%) of the included articles were published between 2010 and 2022 (Table S7 in the **Online Supplementary Document**). All included articles had a quality score of five or above, with the majority scoring eight or above (41 articles, 82.0%), indicating a high quality of included articles (Table S8 in the **Online Supplementary Document**).

### Primary open-angle glaucoma

#### Incidence rate

**Figure 3:**
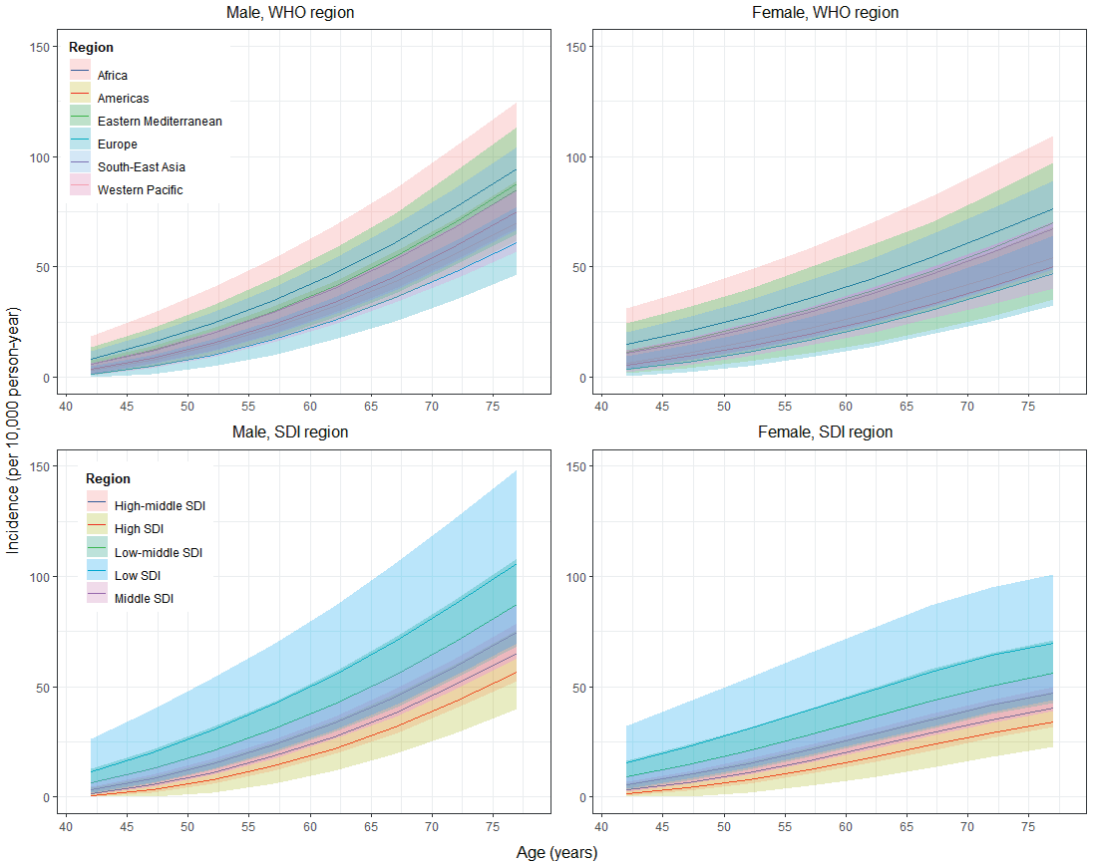
Incidence rate of primary open-angle glaucoma in different World Health Organization regions and social demographic index groups, by age and sex group. SDI – sociodemographic index, WHO – World Health Organization.

On the basis of the extracted and imputed data points (80 in total, 70 extracted and 10 imputed), the age- and sex-specific incidence rates of POAG were estimated (**Table 1**, **Figure 3**). With advanced age, the incidence rate increased from 5.51 (95% CI = 1.63–11.12) per 10 000 person-years among 40–44-year-olds to 64.36 (95% CI = 49.82–80.70) per 10 000 person-years among 75–79-year-olds. The increasing trend was similar in both males and females. However, it became more pronounced in older males aged 55 years and above when compared with their female counterparts. In 2022, the global incidence rate of POAG was 23.46 (95% CI = 15.68–32.91) per 10 000 person-years among 40–79-year-olds. Notably, the incidence rate of POAG in males was comparable to that in females (24.43 (95% CI = 16.59–33.93) per 10 000 person-years vs. 23.93 (95% CI = 14.41–36.19) per 10 000 person-years). Across the six WHO regions, the African region and the European region had the highest and the lowest incidence rates of POAG among males, females, and total population of all age groups. Likewise, the incidence rate was the highest in the low SDI regions and the lowest in high SDI regions along with the age trends (**Figure 3**, Table S9, Table S10 in the **Online Supplementary Document**).

**Table 1:** Estimated incidence rate of primary open-angle glaucoma in adults aged 40–79 y in 2019, by age group and sex (per 10 000 person-years)

Age group	Male	Female	Overall
40–44 y	4.57 (1.06–9.84)	8.44 (2.78–17.15)	5.51 (1.63–11.12)
45–49 y	9.74 (4.52–16.60)	12.74 (5.67–22.55)	10.29 (4.92–17.29)
50–54 y	16.51 (9.67–24.95)	17.97 (9.54–29.00)	16.33 (9.54–24.74)
55–59 y	24.85 (16.40–34.92)	24.02 (14.15–36.49)	23.63 (15.40–33.48)
60–64 y	34.76 (24.64–46.52)	30.93 (19.58–44.91)	32.14 (22.42–43.48)
65–69 y	46.23 (34.37–59.75)	38.60 (25.88–53.91)	41.82 (30.57–54.74)
70–74 y	59.25 (45.58–74.63)	46.85 (32.41–64.01)	52.58 (39.73–67.16)
75–79 y	73.82 (58.23–91.18)	56.16 (39.97–75.17)	64.36 (49.82–80.70)
Overall (40–79 y)	24.43 (16.59–33.93)	23.93 (14.41–36.19)	23.46 (15.68–32.91)

#### Annual cumulative incidence

**Figure 4:**
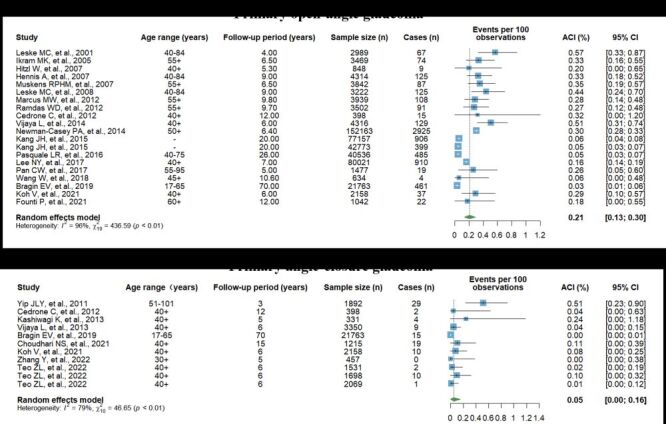
The pooled annual cumulative incidence of primary open-angle glaucoma and primary angle-closure glaucoma. Articles by Teo ZL et al. had different inclusion and exclusion criteria. ACI – annual cumulative incidence, CI – confidence interval.

A total of 42 articles reported the cumulative incidence of POAG, with 14 articles excluded due to missing follow-up time and nine articles excluded for duplicate study populations. This left 19 articles included in meta-analysis with the follow-up period ranging from four to 70 years. The ACI of POAG ranged from 0.26% to 0.57% [31–39]. The pooled ACI of POAG was 0.21% (95% CI = 0.13%–0.30%) (**Figure 4**). Although the heterogeneity test showed heterogeneity among the included articles (*I^2^* = 96%, *P* < 0.01), sensitivity analysis did not reveal any particular article largely affecting the reliability and stability of the pooled ACI of POAG (Figure S1 in the **Online Supplementary Document**). Funnel plots, Egger's regression test, and Begg's rank correlation test did not reveal publication bias (Figure S2 in the **Online Supplementary Document**).

#### Risk factors

**Figure 5:**
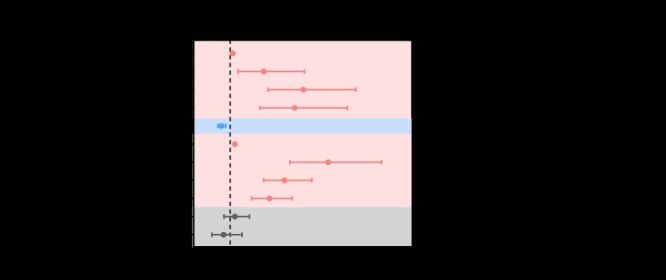
Synthesised effect size of risk factors for primary open-angle glaucoma investigated in at least three articles using multivariable regression. IOP – intraocular pressure, OR – odds ratio, RF – risk factor.

A total of 37 articles identified potential risk factors for POAG employing multivariable regression. Among these, eight potential risk factors for POAG were included in the meta-analysis for risk factors for POAG (**Figure 5**). These included age (per year increase, 50–59, 60–69, and 70 and above age groups), sex, IOP (per one millimetres of mercury (mm Hg) increase), IOP treatment, family history of glaucoma, myopia, diabetes, and hypertension, with at least three data points each. Advanced age was a significant risk factor for POAG, with the risk increasing by 1.07 times for each additional year (meta-OR = 1.07; 95% CI = 1.05–1.08). When compared to the 40–49 age group, the 50–59, 60–69, and 70 and above age groups had increased risks of POAG by 0.92, 2.01, and 1.77 times, respectively (meta-OR = 1.92, 95% CI = 1.22–3.04; meta-OR = 3.01, 95% CI = 2.04–4.45; meta-OR = 2.77, 95% CI = 1.82–4.22). Females had a lower risk of POAG compared to males, with a meta-OR of 0.76 (95% CI = 0.66–0.88). Elevated IOP was identified as one of the risk factors for glaucoma, with the risk of POAG increasing by 1.13 times for every one mmHg increase in IOP (meta-OR = 1.13; 95% CI = 1.11–1.15). IOP treatment was also significantly associated with a higher risk of POAG (meta-OR = 3.69; 95% CI = 2.64–5.15). A positive family history of glaucoma was significantly associated with a higher risk of POAG, with a meta-OR of 2.49 (95% CI = 1.92–3.24). Additionally, the risk of POAG was elevated in individuals with myopia (meta-OR = 2.08; 95% CI = 1.59–2.70), while no significant associations were found with diabetes and hypertension. Detailed information on each potential risk factor for POAG is provided in Table S11 in the **Online Supplementary Document**.

We assessed the credibility levels of identified associations based on our classification evaluation criteria (Table S4 in the **Online Supplementary Document**). Based on the results of the evidence assessment (**Figure 5**), two factors showed strong evidence (IOP, family history of glaucoma); one showed suggestive evidence (female), and three showed weak evidence (age, IOP treatment, myopia) on the association with POAG risk.

### Primary angle-closure glaucoma

#### Cumulative incidence

Ten articles reported the cumulative incidence of PACG, of which nine articles were included in the meta-analysis. The excluded article reported a cumulative incidence of PACG as 0.14% among Russian nuclear workers aged 17–65, followed from 1948 to 31 December 2018 [40]. The meta-analysis showed the pooled ACI of PACG was 0.05% (95% CI = 0.00%–0.16%) (**Figure 4**). Leave-one-out sensitivity analysis demonstrated that the pooled estimates were relatively robust, fluctuating between 0.02% and 0.07% (Figure S3 in the **Online Supplementary Document**). Funnel plots, Egger's regression test, and Begg's rank correlation test did not indicate publication bias (Figure S4 in the **Online Supplementary Document**).

#### Risk factors

Three articles conducted multivariable regression to identify potential risk factors for PACG. However, due to the insufficient number of effective data points, meta-analyses could not be performed. A study based on the Russian Mayak Production Association nuclear workers' cohort explored the relationship between the risk of new-onset PACG among nuclear workers and exposure to gamma or neutron radiation but found no significant association. Gamma radiation exposure, with a mean dose of 1.19 Gray (Gy), was associated with a RR of 0.85 (95% CI = 0.21–2.69). And a neutron radiation exposure with a mean dose of 0.004 Gy, was linked with a RR of 6.60 (95% CI = 0.23–225.00) [40]. In a study involving participants aged 40 and above in rural southern India, it was discovered that female sex was associated with an increased risk of PACG (OR = 2.72; 95% CI = 1.91–3.86), while myopia was linked to a decreased risk (OR = 0.54; 95% CI = 0.35–0.85) [41]. Another cohort study conducted in Singapore among the Indian population aged 40–80 highlighted that a larger cup-to-disc ratio (per 0.1, OR = 2.29; 95% CI = 1.59–3.31), lower socioeconomic status (OR = 7.02; 95% CI = 1.64–29.94), higher body mass index (per unit, OR = 1.06; 95% CI = 1.01–1.11), and hyperopia (OR = 3.17; 95% CI = 1.10–9.09) to be associated with an elevated risk of PACG [11].

### Secondary glaucoma

#### Cumulative incidence

One article reported the cumulative incidence of secondary glaucoma. The British Ophthalmic Surveillance Unit actively monitored new diagnoses of secondary glaucoma in all children aged 16 and below in the United Kingdom. They conducted a follow-up of 1 354 574 newborns from 1 December 2001 to 30 November 2002, and identified 52 new cases of secondary glaucoma, resulting in a cumulative incidence of 0.004% [42].

## DISCUSSION

Based on the comprehensive systematic review and meta-analysis of existing evidence on prospective studies, this study presented estimates of global incidence and risk factors for glaucoma and its subtypes. From 1990 to 2022, the incidence rate and pooled ACI of POAG was 23.46 per 10 000 person-years and 0.21%, respectively. The pooled ACI of PACG was 0.05%. Furthermore, this study identified six potential risk factors for POAG, including advanced age, male, elevated IOP, IOP treatment, family history of glaucoma, and myopia.

POAG accounted for the largest proportion among all the subtypes of glaucoma, with notable regional variations in disease burden. Pooling the evidence from studies on the incidence of POAG based on WHO regions, this study found that the incidence rate in the Africa region was higher than that in other regions, such as Americas and European regions. Our result was to some extent consistent with the regional differences in POAG prevalence reported by Tham et al., which showed that the POAG prevalence was relatively higher in Africa [2]. This regional disparity may be linked to intrinsic physiological differences, such as shorter trabecular meshwork height and thinner central corneal thickness among Africans [43,44], alongside environmental factors such as high temperatures in Africa [45]. In addition, this could also be due to the differences in development levels among regions, which was in accordance with our findings of association between lower SDI and higher risks of POAG. It is widely accepted that lower SDI regions may have a population with lower health literacy and poorer knowledge of glaucoma, as well as limited eye care resources [46].

Meanwhile, this study observed that the ACI of PACG was lower than that of POAG, which was in accordance with the previous estimate of prevalence at the global and national levels [2,47]. Nonetheless, the proportionate vision loss and blindness in both eyes of PACG was proved to be twice as severe as POAG, warranting increased public health concern [15,48,49]. Evidence from previous studies showed that PACG had a greater prevalence in the population of Asia than that in other regions, and accounted for more than half of all cases worldwide [2,50–52]. Since most of the studies included in this study were conducted in Asia rather than in other regions, such as the Americas, further comparison of PACG incidence in different regions could not be made yet.

Secondary glaucoma is mainly caused by several ocular or systemic disorders, which might be more preventable compared to primary glaucoma [53]. So far, the epidemiological data on secondary glaucoma has been limited and only focused on the unrepresentative sample population, such as hospital-based analysis [54,55]. Therefore, population-based epidemiological studies on the profile of secondary glaucoma are warranted in the future.

This study identified six risk factors for POAG, including advanced age, male, high IOP, IOP treatment, family history of glaucoma, and myopia. Consistent with previous studies [32,56–58], the incidence rate of POAG observed in this study showed an increasing trend with advanced age at both global and regional levels. Since hospital visits were often made after patients developed visual impairment, the onset of glaucoma tended to be much earlier than the time of diagnosis. As a neurodegenerative disease, there might be a fair chance of an alarming increase in the disease burden of glaucoma with increasing life expectancy worldwide, especially primary glaucoma [59]. Therefore, attention should be paid to the early screening of glaucoma, as well as health education among elderly people. Moreover, in our study, the incidence rate of POAG in males was comparable to that in females. Evidence from the Ponza eye study in Italy showed increased susceptibility to POAG in males, whereas, no difference was found in Bai Chinese [12,56]. The inconsistent results from the above studies could be ascribed to the heterogeneity of regional environment and study population. It follows that more prospective studies are needed to further explore sex differences in glaucoma and their related mechanism in the future.

Elevated IOP is one of the most important risk factors for POAG that could be controlled with medical treatment or surgery [15,60]. A 12-year follow-up study of an Italian population revealed that individuals with IOP≥22 mm Hg at baseline had a 7-fold increased risk of developing POAG [56]. Consistently, elevated IOP was identified as one of the risk factors for glaucoma in this study, with the risk of POAG increasing by 0.13 times for every one mmHg increase in IOP. In contrast, there was still a large proportion of POAG cases without evidence of elevated IOP (normal tension glaucoma), which emphasised that the diagnosis of POAG should also be based on the optic disc and visual field in addition to IOP examination [61]. The association between elevated IOP and the risk of glaucoma might also be influenced by other factors. For instance, the optic nerve head could gradually become more susceptible to the change of IOP with aging [12]. However, this study observed that IOP treatment was associated with higher risks of POAG. Surgical methods for elevated IOP such as trabeculectomy were found a significant link with more rapid progression of glaucoma [62]. This could be partly explained by more prior and advanced treatment for individuals with more severe glaucoma, as well as delay in diagnosis when the irreversible visual impairment has already occurred [63]. Besides, studies have shown that latanoprost, as one of the ocular hypotensive medications, was likely to increase trabecular meshwork contractility and lead to the development of glaucoma [64].

Alternatively, it was found in our analysis that a positive family history of glaucoma was associated with higher risks of POAG. Previous studies suggested that genetics plays a part in the incidence of primary glaucoma with an estimated heritability of 70% [65,66]. Therefore, regular examination of the optic disc and visual field should be done among individuals with a family history of glaucoma. However, in most cases (over 90%) POAG arose from both genetic and environmental factors, rather than just explained by genetic effect [67,68].

Moreover, we investigated the link between myopia and POAG risks, which showed that myopia might double the risk of developing POAG. Similar patterns of associations were noted in prospective studies from China, Netherlands, and Italy [12,32,56]. Several mechanisms have been put forward to explain the above association, including higher susceptibility of the optic nerve head to glaucomatous damage from elevated IOP and the shearing forces exerted by scleral tension [69]. Besides, previous studies demonstrated that diabetes could increase the risk of POAG by neuronal injury from stress and reduced blood flow to the retina and optic nerve [70,71], which was not observed in this study. This could be the result of more frequent ophthalmologic visits among diabetes patients, as well as the limitation that we were unable to distinguish among classifications of diabetes based on current evidence [72]. Likewise, the association between hypertension and POAG was not found in this analysis. Some studies showed a positive connection of IOP with blood pressure and POAG, but the relation between BP and POAG remained controversial [73]. More and more evidence has recently come to reveal that ocular perfusion pressure, a composite reflection of BP and IOP, was more significantly related to the risk of POAG. For instance, a lower BP in the context of higher IOP might lead to poor optic nerve perfusion, and further give rise to the development of POAG [74,75].

This study has several strengths as follows. To the best of our knowledge, our study is the most comprehensive systematic review and meta-analysis to report the global incidence and risk factors for glaucoma and its subtypes, adding new evidence to the epidemiological studies of glaucoma. Additionally, the full-scale search strategy along with rigorous inclusion and exclusion criteria were adopted for literature screening, which ensured the comprehensiveness and reliability of the current study. Moreover, only those prospective studies reporting ORs of risk factors through multivariable analyses were included in the meta-analysis, thereby mitigating the underlying biases associated with univariable analyses.

It should be acknowledged that our study also has certain limitations. First, there was heterogeneity among the included articles concerning study design, target population, and methodology. Nonetheless, we did not find any single study that had an impact on the effect size in the sensitivity analysis, ensuring the reliability and robustness of our work. Second, exclusion based on glaucoma definition, grading system, and examination method was not conducted in the literature screening of our study. Previous studies have shown consistency of glaucoma prevalence rates using different diagnostic criteria and investigation methods, whereas it might still be influenced by the inherent subjectivity of diagnosis and not necessarily applicable to the study of glaucoma incidence [47]. What’s more, the current study did not further explore the risk factor profile of other glaucoma subtypes like PACG and secondary glaucoma, as well as other factors including race, smoking, and drinking, due to the limited research evidence. Therefore, more epidemiological data on the incidence and risk factors of glaucoma are needed in the future.

## CONCLUSIONS

This systematic review and meta-analysis have revealed the global incidence of glaucoma, which is indeed worthy of attention considering the global trend of population ageing. We also identified underlying risk factors associated with the development of glaucoma, calling for public health interventions tailored for people of different ages, regions, and so forth. Additionally, more elaborate epidemiological evidence is needed to better evaluate the disease burden of glaucoma across various regions, especially less-developed and resource-limited areas, and to provide a basis for the health care resource allocation promoting eye health.

## Additional material


Online Supplementary Document
