# Supplementary material for: Global incidence and risk factors for glaucoma: A systematic review and meta-analysis of prospective studies
Source: J Glob Health. 2024 Nov 8;14:04252. doi: 10.7189/jogh.14.04252 (PMC11544525; doi:10.7189/jogh.14.04252)
Supplement: Online Supplementary Document [file jogh-14-04252-s001.pdf]

Supplementary Appendix

Shan et al. Global incidence and risk factors for glaucoma: a systematic review and meta-analysis of prospective studies

This supplementary material has been provided by the authors to give readers additional information about their work.

Contents

Table S1. Search strategy 3
Table S2. The Newcastle-Ottawa Quality Assessment Scale for cohort studies 5
Table S3. meta-regression models of cluster-level factors related to the incidence rate of primary open-angle glaucoma 6
Table S4. Evidence credibility grading criteria 7
Table S5. Full list of the included articles (n=50) 8
Table S6. Detailed characteristics of the included articles (n=50) 13
Table S7. Main characteristics of the included articles 32
Table S8. Quality assessment of included articles (n=50) 35
Table S9. Estimated incidence rate of primary open-angle glaucoma in adults aged 40 – 79 years in 2019, by WHO region (per 10,000 person-years) 38
Table S10. Estimated incidence rate of primary open-angle glaucoma in adults aged 40 – 79 years in 2019, by SDI region (per 10,000 person-years) 40
Table S11. Meta-analyses of risk factors for primary open-angle glaucoma 42
Figure S1. Leave-one-out sensitivity analysis for the pooled annual cumulative incidence of primary open-angle glaucoma. 46
Figure S2. Publication bias test for the pooled annual cumulative incidence of primary open-angle glaucoma. 47
Figure S3. Leave-one-out sensitivity analysis for the pooled annual cumulative incidence of primary angle-closure glaucoma. 48
Figure S4. Publication bias test for the pooled annual cumulative incidence of primary angle-closure glaucoma. 49

Table S1. Search strategy

Database	Date	Search Strategy
**CNKI**	29th Nov, 2022	SU% '青光眼' AND (SU% '发病率'+'发生率'+'死亡率'+'病死率'+'流行') AND (FT% '调查'+'监测'+'前瞻'+'队列'+'随访')
**Wanfang**	29th Nov, 2022	(主题:(青光眼) and (主题:(发病率) or 主题:(发生率) or 主题:(死亡率) or 主题:(病死率) or 主题:(流行)) and (全部:(调查) or 全部:(监测) or 全部:(前瞻) or 全部:(队列) or 全部:(随访))) and Date:1990-*
**CQVIP**	29th Nov, 2022	(M=青光眼 OR R=青光眼) AND (M=(发病率 OR 发生率 OR 死亡率 OR 病死率 OR 流行) OR R=(发病率 OR 发生率 OR 死亡率 OR 病死率 OR 流行)) AND U=(调查 OR 监测 OR 前瞻 OR 队列 OR 随访)
**PubMed**	29th Nov, 2022	"glaucoma*"[Title/Abstract] AND ("new-onset"[Title/Abstract] OR "inciden*"[Title/Abstract] OR "morbidit*"[Title/Abstract] OR "mortalit*"[Title/Abstract] OR "fatalit*"[Title/Abstract] OR "death"[Title/Abstract] OR "epidemiolog*"[Title/Abstract]) AND ("cohort*"[All Fields] OR "prospective"[All Fields] OR "follow-up*"[All Fields] OR "longitudinal"[All Fields]) AND 1990/01/01:2022/11/29[Date - Publication] AND "humans"[MeSH Terms]
**Embase**	29th Nov, 2022	1 'glaucoma'/exp OR 'glaucoma*':ab,ti<br>2 'new-onset':ab,ti<br>3 'incidence'/exp OR 'inciden*':ab,ti<br>4 'morbidity'/exp OR 'morbidit*':ab,ti<br>5 'mortality'/exp OR 'mortalit*':ab,ti<br>6 'fatality'/exp OR 'fatalit*':ab,ti<br>7 'death'/exp OR 'death':ab,ti<br>8 'epidemiology'/exp OR 'epidemiolog*':ab,ti<br>9 #2 OR #3 OR #4 OR #5 OR #6 OR #7 OR #8<br>10 'cohort analysis'/exp OR 'cohort*'<br>11 'prospective study'/exp OR 'prospective'<br>12 'follow up'/exp OR 'follow-up*'<br>13 'longitudinal study'/exp OR 'longitudinal'<br>14 #10 OR #11 OR #12 OR #13<br>15 #1 AND #9 AND #14<br>16 #15 AND [humans]/lim AND [1990-2022]/py<br>17 #16 AND [embase]/lim NOT ([embase]/lim AND [medline]/lim)

Database	Date	Search Strategy
**Medline**	29th Nov, 2022	1 exp Glaucoma/ or glaucoma*.ab,ti.<br>2 New-onset.ab,ti.<br>3 exp Incidence/ or Inciden*.ab,ti.<br>4 exp Morbidity/ or Morbidit*.ab,ti.<br>5 exp Mortality/ or Mortalit*.ab,ti.<br>6 fatalit*.ab,ti.<br>7 exp Death/ or Death.ab,ti.<br>8 exp Epidemiology/ or Epidemiolog*.ab,ti.<br>9 2 or 3 or 4 or 5 or 6 or 7 or 8<br>10 exp Cohort Studies/ or cohort*.af.<br>11 exp Prospective studies/ or Prospective.af.<br>12 exp Follow-Up Studies/ or Follow-up*.af.<br>13 exp Longitudinal studies/ or Longitudinal.af.<br>14 10 or 11 or 12 or 13<br>15 1 and 9 and 14<br>16 limit 15 to (humans and yr="1990 -Current")

Table S2. The Newcastle-Ottawa Quality Assessment Scale for cohort studies

---

Selection

1) Representativeness of the exposed cohort

a) truly representative of the average _______________ (describe) in the community ※

b) somewhat representative of the average ______________ in the community ※

c) selected group of users eg nurses, volunteers

d) no description of the derivation of the cohort

2) Selection of the non exposed cohort

a) drawn from the same community as the exposed cohort ※

b) drawn from a different source

c) no description of the derivation of the non exposed cohort

3) Ascertainment of exposure

a) secure record (eg surgical records) ※

b) structured interview ※

c) written self report

d) no description

4) Demonstration that outcome of interest was not present at start of study

a) yes ※

b) no

Comparability

1) Comparability of cohorts on the basis of the design or analysis

a) study controls for _____________ (select the most important factor) ※

b) study controls for any additional factor ※ (This criteria could be modified to indicate specific control for a second important factor.)

Outcome

1) Assessment of outcome

a) independent blind assessment ※

b) record linkage ※

c) self report

d) no description

2) Was follow-up long enough for outcomes to occur

a) yes (select an adequate follow up period for outcome of interest-3 years) ※

b) no

3) Adequacy of follow up of cohorts

a) complete follow up - all subjects accounted for ※

b) subjects lost to follow up unlikely to introduce bias - small number lost - > 70 % (select an adequate %) follow up, or description provided of those lost) ※

c) follow up rate < 70 % (select an adequate %) and no description of those lost

d) no statement

Notes: A study can be awarded a maximum of one star for each numbered item within the Selection and Outcome categories. A maximum of two stars can be given for Comparability.

Table S3. meta-regression models of cluster-level factors related to the incidence rate of primary open-angle glaucoma

Moderator	Number of articles	Number of data points	β	95 % CI		*P* value
**Age**	23	71	0.0016	0.0015	0.0017	< .0001
**Female proportion**	27	74	-0.0015	-0.0025	-0.006	0.0018
**WHO region**						
**AMR**	22	29	Reference			
**EUR**	9	25	-0.0011	-0.0258	0.0235	0.9274
**SEAR**	1	8	0.0231	-0.0147	0.0610	0.2308
**WPR**	3	12	0.0016	-0.0259	0.0291	0.9091
**SDI**	29	80	0.0312	-0.0415	0.1040	0.4002

Notes: CI, confidence interval; SDI, Socio-demographic Index; AMR=Region of the Americas, EUR, European Region; SEAR, South-East Asian Region; WPR, Western Pacific Region.

Table S4. Evidence credibility grading criteria

Category	Criteria
Convincing evidence (class I)	● P-value < 1×10^{-6}<br>● More than 1000 cases<br>● 95% prediction interval excluding the null value<br>● I^2 < 50%<br>● No small-study effects and excess significance bias
Highly suggestive evidence (class II)	● P-value < 1×10^{-6}<br>● More than 1000 cases<br>●A statistically significant result reported in the largest individual study
Suggestive evidence (class III)	● P-value < 1×10^{-3}<br>● More than 1000 cases
Weak evidence (class IV)	● P-value < 0.05
Non-significant (NS)	● P-value > 0.05

Table S5. Full list of the included articles (n=50)

Article ID	Reference
**G1**	Zhang Y, Zhang Q, Thomas R, Li SZ, Wang NL. Development of angle closure and associated risk factors: The Handan eye study. Acta Ophthalmol. 2022;100(1): e253-e261.
**G2**	Hanyuda A, Rosner BA, Wiggs JL, et al. Prospective study of dietary intake of branched-chain amino acids and the risk of primary open-angle glaucoma. Acta Ophthalmol. 2022;100(3): e760-e769.
**G3**	Teo ZL, Soh ZD, Tham YC, et al. Six-Year Incidence and Risk Factors for Primary Angle-Closure Disease: The Singapore Epidemiology of Eye Diseases Study. Ophthalmology. 2022;129(7):792-802.
**G4**	Azizova TV, Bragin EV, Bannikova MV, Hamada N, Grigoryeva ES. The Incidence Risk for Primary Glaucoma and Its Subtypes following Chronic Exposure to Ionizing Radiation in the Russian Cohort of Mayak Nuclear Workers. Cancers (Basel). 2022;14(3).
**G5**	Choudhari NS, Khanna RC, Marmamula S, et al. Fifteen-Year Incidence Rate of Primary Angle Closure Disease in the Andhra Pradesh Eye Disease Study. Am J Ophthalmol. 2021; 229: 34-44.
**G6**	Bragin EV, Azizova TV, Bannikova MV, Grinyov AG. Incidence of primary glaucoma in a cohort of nuclear workers. Oftalmologiya. 2021;18(3):560-565.
**G7**	Koh V, Tham YC, Tan N, et al. Six-Year Incidence and Risk Factors of Primary Glaucoma in the Singapore Indian Eye Study. Ophthalmol Glaucoma. 2021;4(2):201-208.
**G8**	Founti P, Coleman AL, Wilson MR, et al. Twelve-Year Incidence of Open-angle Glaucoma: The Thessaloniki Eye Study. J Glaucoma. 2021;30(9):851-858.
**G9**	Hanyuda A, Rosner BA, Wiggs JL, et al. Low-carbohydrate-diet scores and the risk of primary open-angle glaucoma: data from three US cohorts. Eye (Lond). 2020;34(8):1465-1475.
**G10**	Jung Y, Han K, Park H, Lee SH, Park CK. Metabolic Health, Obesity, and the Risk of Developing Open-Angle Glaucoma: Metabolically Healthy Obese Patients versus Metabolically Unhealthy but Normal Weight Patients. Diabetes Metab J. 2020;44(3):414-425.
**G11**	Bragin EV, Azizova TV, Bannikova MV, Grigoryeva ES, Hamada N. Glaucoma incidence risk in a cohort of Mayak PA workers occupationally exposed to ionizing radiation. Sci Rep. 2019;9(1):12469.

Article ID	Reference
G12	Wang W, Moroi S, Bakulski K, et al. Bone Lead Levels and Risk of Incident Primary Open-Angle Glaucoma: The VA Normative Aging Study. Environ Health Perspect. 2018;126(8):87002.
G13	Kang JH, Ivey KL, Boumenna T, Rosner B, Wiggs JL, Pasquale LR. Prospective study of flavonoid intake and risk of primary open-angle glaucoma. Acta Ophthalmol. 2018;96(6): e692-e700.
G14	Lee NY, Jung Y, Han K, Park CK. Fluctuation in systolic blood pressure is a major systemic risk factor for development of primary open-angle glaucoma. Sci Rep. 2017; 7: 43734.
G15	Pan CW, Yang WY, Hu DN, et al. Longitudinal Cohort Study on the Incidence of Primary Open-Angle Glaucoma in Bai Chinese. Am J Ophthalmol. 2017; 176: 127-133.
G16	Kang JH, Willett WC, Rosner BA, Buys E, Wiggs JL, Pasquale LR. Association of Dietary Nitrate Intake With Primary Open-Angle Glaucoma: A Prospective Analysis From the Nurses' Health Study and Health Professionals Follow-up Study. JAMA Ophthalmol. 2016;134(3):294-303.
G17	Li L, Li C, Zhong H, Tao Y, Yuan Y, Pan CW. Estimated Cerebrospina Fluid Pressure and the 5-Year Incidence of Primary Open-Angle Glaucoma in a Chinese Population. PLoS One. 2016;11(9): e162862.
G18	Pasquale LR, Hyman L, Wiggs JL, et al. Prospective Study of Oral Health and Risk of Primary Open-Angle Glaucoma in Men: Data from the Health Professionals Follow-up Study. Ophthalmology. 2016;123(11):2318-2327.
G19	Kang JH, Loomis SJ, Rosner BA, Wiggs JL, Pasquale LR. Comparison of Risk Factor Profiles for Primary Open-Angle Glaucoma Subtypes Defined by Pattern of Visual Field Loss: A Prospective Study. Invest Ophthalmol Vis Sci. 2015;56(4):2439-2448.
G20	Vijaya L, Rashima A, Panday M, et al. Predictors for incidence of primary open-angle glaucoma in a South Indian population: the Chennai eye disease incidence study. Ophthalmology. 2014;121(7):1370-1376.
G21	Newman-Casey PA, Talwar N, Nan B, Musch DC, Pasquale LR, Stein JD. The potential association between postmenopausal hormone use and primary open-angle glaucoma. JAMA Ophthalmol. 2014;132(3):298-303.
G22	Kashiwagi K, Chiba T, Mabuchi F, Furuya T, Tsukahara S. Five-year incidence of angle closure among glaucoma health examination participants. Graefes Arch Clin Exp Ophthalmol. 2013;251(4):1219-1228.
G23	Vijaya L, Asokan R, Panday M, et al. Six-year incidence of angle-closure disease in a South Indian population: the Chennai Eye Disease Incidence Study. Am J Ophthalmol. 2013;156(6):1308-1315.

Article ID	Reference
**G24**	Marcus MW, Müskens RP, Ramdas WD, et al. Antithrombotic medication and incident open-angle glaucoma. Invest Ophthalmol Vis Sci. 2012;53(7):3801-3805.
**G25**	Marcus MW, Müskens RP, Ramdas WD, et al. Cholesterol-lowering drugs and incident open-angle glaucoma: a population-based cohort study. PLoS One. 2012;7(1): e29724.
**G26**	Marcus MW, Müskens RP, Ramdas WD, et al. Corticosteroids and open-angle glaucoma in the elderly: a population-based cohort study. Drugs Aging. 2012;29(12):963-970.
**G27**	Ramdas WD, Wolfs RC, Kiefte-de JJ, et al. Nutrient intake and risk of open-angle glaucoma: the Rotterdam Study. Eur J Epidemiol. 2012;27(5):385-393.
**G28**	Cedrone C, Mancino R, Ricci F, Cerulli A, Culasso F, Nucci C. The 12-year incidence of glaucoma and glaucoma-related visual field loss in Italy: the Ponza eye study. J Glaucoma. 2012;21(1):1-6.
**G29**	Wise LA, Rosenberg L, Radin RG, et al. A prospective study of diabetes, lifestyle factors, and glaucoma among African-American women. Ann Epidemiol. 2011;21(6):430-439.
**G30**	Pasquale LR, Kang JH. Female reproductive factors and primary open-angle glaucoma in the Nurses' Health Study. Eye (Lond). 2011;25(5):633-641.
**G31**	Yip JLY, Nolan WP, Davaatseren U, et al. Primary angle closure glaucoma in East Asia: educational attainment as a protective factor. Ophthalmic Epidemiol. 2011;18(5):217-225.
**G32**	Pasquale LR, Willett WC, Rosner BA, Kang JH. Anthropometric measures and their relation to incident primary open-angle glaucoma. Ophthalmology. 2010;117(8):1521-1529.
**G33**	Kang JH, Willett WC, Rosner BA, Hankinson SE, Pasquale LR. Caffeine consumption and the risk of primary open-angle glaucoma: a prospective cohort study. Invest Ophthalmol Vis Sci. 2008;49(5):1924-1931.
**G34**	Leske MC, Wu S, Hennis A, Honkanen R, Nemesure B, BESs SG. Risk factors for incident open-angle glaucoma: the Barbados Eye Studies. Ophthalmology. 2008;115(1):85-93.
**G35**	Hitzl W, Hornykewycz K, Grabner G, Reitsamer HA. [On the relationship between age and prevalence and/or incidence of primary open-angle glaucoma in the "Salzburg-Moorfields Collaborative Glaucoma Study"]. Klin Monbl Augenheilkd. 2007;224(2):115-119.
**G36**	Hennis A, Wu S, Nemesure B, Honkanen R, Leske MC, Barbados ESG. Awareness of incident open-angle glaucoma in a population study: the Barbados Eye Studies. Ophthalmology. 2007;114(10):1816-1821.

Article ID	Reference
**G37**	Nemesure B, Honkanen R, Hennis A, Wu SY, Leske MC, Barbados ESG. Incident open-angle glaucoma and intraocular pressure. Ophthalmology. 2007;114(10):1810-1815.
**G38**	Leske MC, Wu SY, Honkanen R, et al. Nine-year incidence of open-angle glaucoma in the Barbados Eye Studies. Ophthalmology. 2007;114(6):1058-1064.
**G39**	Muskens RPHM, de Voogd S, Wolfs RCW, et al. Systemic antihypertensive medication and incident open-angle glaucoma. Ophthalmology. 2007;114(12):2221-2226.
**G40**	Papadopoulos M, Cable N, Rahi J, Khaw PT, BIG ESI. The British Infantile and Childhood Glaucoma (BIG) Eye Study. Invest Ophthalmol Vis Sci. 2007;48(9):4100-4106.
**G41**	de Voogd S, Wolfs RCW, Jansonius NM, Witteman JCM, Hofman A, de Jong PTVM. Atherosclerosis, C-reactive protein, and risk for open-angle glaucoma: the Rotterdam study. Invest Ophthalmol Vis Sci. 2006;47(9):3772-3776.
**G42**	de Voogd S, Ikram MK, Wolfs RCW, et al. Is diabetes mellitus a risk factor for open-angle glaucoma? The Rotterdam Study. Ophthalmology. 2006;113(10):1827-1831.
**G43**	Pasquale LR, Kang JH, Manson JE, Willett WC, Rosner BA, Hankinson SE. Prospective study of type 2 diabetes mellitus and risk of primary open-angle glaucoma in women. Ophthalmology. 2006;113(7):1081-1086.
**G44**	de Voogd S, Ikram MK, Wolfs RCW, Jansonius NM, Hofman A, de Jong PTVM. Incidence of open-angle glaucoma in a general elderly population: the Rotterdam Study. Ophthalmology. 2005;112(9):1487-1493.
**G45**	Ikram MK, de Voogd S, Wolfs RCW, et al. Retinal vessel diameters and incident open-angle glaucoma and optic disc changes: the Rotterdam study. Invest Ophthalmol Vis Sci. 2005;46(4):1182-1187.
**G46**	Sloan FA, Brown DS, Carlisle ES, Ostermann J, Lee PP. Estimates of incidence rates with longitudinal claims data. Archives of ophthalmology (Chicago, Ill. : 1960). 2003;121(10):1462-1468.
**G47**	Nemesure B, Wu S, Hennis A, Leske MC, Barbados ESG. Factors related to the 4-year risk of high intraocular pressure: the Barbados Eye Studies. Archives of ophthalmology (Chicago, Ill. : 1960). 2003;121(6):856-862.
**G48**	Kang JH, Pasquale LR, Rosner BA, et al. Prospective study of cigarette smoking and the risk of primary open-angle glaucoma. Archives of ophthalmology (Chicago, Ill. : 1960). 2003;121(12):1762-1768.

Article ID	Reference
**G49**	Leske MC, Wu S, Nemesure B, Hennis A. Incident open-angle glaucoma and blood pressure. Archives of ophthalmology (Chicago, Ill. : 1960). 2002;120(7):954-959.
**G50**	Leske MC, Connell AM, Wu SY, et al. Incidence of open-angle glaucoma: the Barbados Eye Studies. The Barbados Eye Studies Group. Arch Ophthalmol. 2001;119(1):89-95.

Table S6. Detailed characteristics of the included articles (n=50)

Article ID	Author	Publish year	Country	WHO region	SDI region	Urban/ Rural	Baseline	Follow-up time	ACA/ depth evaluation	IOP measurement	Optic disc evaluation	Visual field testing	Visual acuity	Age range (year)	Female proportion	Incidence type	Sample	POAG	PACG	Secondary glaucoma
**G1**	Zhang Y, et al.	2022	China	WPR	M-SDI	Rural	2006	5 years	Yes, suspect	Yes, all	Yes, all	Yes, all	Yes, all	30+	0.65	CI	457	-	0	-
**G2**	Hanyuda A, et al.	2022	United states	AMR	H-SDI	Mixed	1986	NA	Yes, all	Yes, all	Yes, all	Yes, all	No	40-75	0	CI	42254	560	-	-
**G2**	Hanyuda A, et al.	2022	United states	AMR	H-SDI	Mixed	1984 (NHS), 1991 (NHS II)	NA	Yes, all	Yes, all	Yes, all	Yes, all	No	40-55	1	CI	132435	1386	-	-
**G2**	Hanyuda A, et al.	2022	United states	AMR	H-SDI	Mixed	1984 (NHS), 1991 (NHS	NA	Yes, all	Yes, all	Yes, all	Yes, all	No	40-75	75.8	IR	3109037	1946	-	-

Article ID	Author	Publish year	Country	WHO region	SDI region	Urban/ Rural	Baseline	Follow-up time	ACA/ depth evaluation	IOP measurement	Optic disc evaluation	Visual field testing	Visual acuity	Age range (year)	Female proportion	Incidence type	Sample	POAG	PACG	Secondary glaucoma
							II), 1986 (HPFS)													
**G3**	Teo ZL, et al.	2022	Singapore	WPR	H-SDI	Mixed	2004-2006	6 years	Yes, all	Yes, all	Yes, all	Yes, all	No	40+	NA	CI	1531	-	2	-
**G3**	Teo ZL, et al.	2022	Singapore	WPR	H-SDI	Mixed	2007-2009	6 years	Yes, all	Yes, all	Yes, all	Yes, all	No	40+	NA	CI	1698	-	10	-
**G3**	Teo ZL, et al.	2022	Singapore	WPR	H-SDI	Mixed	2009-2011	6 years	Yes, all	Yes, all	Yes, all	Yes, all	No	40+	NA	CI	2069	-	1	-
**G4**	Azizova TV, et al.	2022	Russia	EUR	M-SDI	Mixed	1948-1982	NA	Yes, all	Yes, all	Yes, all	Yes, all	No	17-65	NA	CI	21650	540	32	-
**G5**	Choudhari NS, et al.	2021	India	SEAR	LM -	Rural	1996-2000	15 years	Yes, all	Yes, suspect	Yes, all	Yes ,	Yes, all	40+	0.529	CI	1215	-	19	-

Article ID	Author	Publish year	Country	WHO region	SDI region	Urban/ Rural	Baseline	Follow-up time	ACA/ depth evaluation	IOP measurement	Optic disc evaluation	Visual field testing	Visual acuity	Age range (year)	Female proportion	Incidence type	Sample	POAG	PACG	Secondary glaucoma
					SDI							suspect								
**G6**	Bragin EV, et al.	2021	Russia	EUR	LM-SDI	Mixed	1948-1982	NA	Yes, all	Yes, all	Yes, all	Yes, all	No	NA	NA	IR	482217	476	-	-
**G7**	Koh V, et al.	2021	Singapore	WPR	H-SDI	Mixed	2007-2009	6 years	Yes, all	Yes, all	Yes, all	Yes, suspect	Yes, all	40+	NA	CI	2158	37	10	-
**G8**	Founti P, et al.	2021	Greece	EUR	HM-SDI	Urban	2000-2005	12 years	Yes, all	Yes, all	Yes, all	Yes, all	Yes, all	60+	0.48	CI	1042	22	-	-
**G9**	Hanyuda A, et al.	2020	United states	AMR	HM-SDI	Mixed	1980	NA	Yes, all	Yes, all	Yes, all	Yes, all	No	30-55	1	CI	78210	1357	-	-

Article ID	Author	Publish year	Country	WHO region	SDI region	Urban/ Rural	Baseline	Follow-up time	ACA/ depth evaluation	IOP measurement	Optic disc evaluation	Visual field testing	Visual acuity	Age range (year)	Female proportion	Incidence type	Sample	POAG	PACG	Secondary glaucoma
**G9**	Hanyuda A, et al.	2020	United states	AMR	H-SDI	Mixed	1991	NA	Yes, all	Yes, all	Yes, all	Yes, all	No	24-44	1	CI	66350	217	-	-
**G9**	Hanyuda A, et al.	2020	United states	AMR	HM-SDI	Mixed	1986	NA	Yes, all	Yes, all	Yes, all	Yes, all	No	40-75	0	CI	41078	538	-	-
**G9**	Hanyuda A, et al.	2020	United states	AMR	HM-SDI	Mixed	1980 (NHS), 1986 (HPFS), 1991 (NHS II)	25 years	Yes, all	Yes, all	Yes, all	Yes, all	No	24-75	0.7787	IR	3429825	2112	-	-
**G10**	Jung Y, et al.	2020	the Repub	WPR	H-SDI	Both	2002	NA	No	No	No	No	No	40+	0.509	CI	287553	4970	-	-

Article ID	Author	Publish year	Country	WHO region	SDI region	Urban/ Rural	Baseline	Follow-up time	ACA/ depth evaluation	IOP measurement	Optic disc evaluation	Visual field testing	Visual acuity	Age range (year)	Female proportion	Incidence type	Sample	POAG	PACG	Secondary glaucoma
			lic of Korea																	
**G11**	Bragin EV, et al.	2019	Russia	EUR	M-SDI	Mixed	1948-1982	70 years	Yes, all	Yes, all	Yes, all	Yes, all	No	17-65	0.2541	CI	21763	461	15	-
**G11**	Bragin EV, et al.	2019	Russia	EUR	M-SDI	Mixed	1948-1982	70 years	Yes, all	Yes, all	Yes, all	Yes, all	No	17-65	0.2541	IR	544062	461	-	-
**G12**	Wang W, et al.	2018	United states	AMR	H-SDI	Mixed	1963	10.6 years	No	Yes, all	Yes, all	Yes, all	Yes, all	45+	0	CI	634	4	-	-
**G13**	Kang JH, et al.	2018	United states	AMR	HM-SDI	Mixed	1976	NA	Yes, all	No	Yes, all	Yes, all	No	40-55	1	CI	65516	1058	-	-
**G13**	Kang JH, et al.	2018	United states	AMR	HM-SDI	Mixed	1986	NA	Yes, all	No	Yes, all	Yes, all	No	40-75	0	CI	42156	517	-	-

Article ID	Author	Publish year	Country	WHO region	SDI region	Urban/ Rural	Baseline	Follow-up time	ACA/ depth evaluation	IOP measurement	Optic disc evaluation	Visual field testing	Visual acuity	Age range (year)	Female proportion	Incidence type	Sample	POAG	PACG	Secondary glaucoma
**G13**	Kang JH, et al.	2018	United states	AMR	HM-SDI	Mixed	1976 (NHS), 1986 (HPFS)	NA	Yes, all	No	Yes, all	Yes, all	No	40-75	0.61	IR	1706804	1575	-	-
**G14**	Lee NY, et al.	2017	the Republic of Korea	WPR	H-SDI	Both	2002-2006	7 years	No	No	No	No	No	40+	0.41	CI	80021	910	-	-
**G15**	Pan CW, et al.	2017	China	WPR	M-SDI	Rural	2010	5 years	Yes, all	Yes, all	Yes, all	Yes, suspect	Yes, all	55-95	0.636	CI	1477	19	-	-
**G16**	Kang JH, et al.	2016	United states	AMR	HM-SDI	Mixed	1976	NA	Yes, all	Yes, all	Yes, all	Yes, all	No	40+	1	CI	45955	1000	-	-

Article ID	Author	Publish year	Country	WHO region	SDI region	Urban/ Rural	Baseline	Follow-up time	ACA/ depth evaluation	IOP measurement	Optic disc evaluation	Visual field testing	Visual acuity	Age range (year)	Female proportion	Incidence type	Sample	POAG	PACG	Secondary glaucoma
**G16**	Kang JH, et al.	2016	United states	AMR	HM-SDI	Mixed	1986	NA	Yes, all	Yes, all	Yes, all	Yes, all	No	40+	0	CI	29039	483	-	-
**G16**	Kang JH, et al.	2016	United states	AMR	HM-SDI	Mixed	1976 (NHS), HPFS (1986)	NA	Yes, all	Yes, all	Yes, all	Yes, all	No	40+	0.61	IR	1678713	1483	-	-
**G17**	Li L, et al.	2016	China	WPR	M-SDI	Rural	2010	5 years	Yes, all	Yes, all	Yes, all	Yes, suspect	Yes, all	55-95	NA	CI	1485	19	-	-
**G18**	Pasquale LR, et al.	2016	United states	AMR	HM-SDI	Mixed	1986	26 years	Yes, all	Yes, all	Yes, all	Yes, all	No	40-75	0	CI	40536	485	-	-

Article ID	Author	Publish year	Country	WHO region	SDI region	Urban/Rural	Baseline	Follow-up time	ACA/depth evaluation	IOP measurement	Optic disc evaluation	Visual field testing	Visual acuity	Age range (year)	Female proportion	Incidence type	Sample	POAG	PACG	Secondary glaucoma
**G18**	Pasquale LR, et al.	2016	United states	AMR	HM-SDI	Mixed	1986	26 years	Yes, all	Yes, all	Yes, all	Yes, all	No	40-75	0	IR	528089	485	-	-
**G19**	Kang JH, et al.	2015	United states	AMR	HM-SDI	Mixed	1976	20+ years	Yes, all	Yes, all	Yes, all	Yes, all	No	NA	1	CI	77157	906	-	-
**G19**	Kang JH, et al.	2015	United states	AMR	HM-SDI	Mixed	1986	20+ years	Yes, all	Yes, all	Yes, all	Yes, all	No	NA	0	CI	42773	399	-	-
**G19**	Kang JH, et al.	2015	United states	AMR	HM-SDI	Mixed	1976 (NHS), 1986 (HPFS)	20+ years	Yes, all	Yes, all	Yes, all	Yes, all	No	NA	0.643	IR	1716214	1305	-	-

Article ID	Author	Publish year	Country	WHO region	SDI region	Urban/ Rural	Baseline	Follow-up time	ACA/ depth evaluation	IOP measurement	Optic disc evaluation	Visual field testing	Visual acuity	Age range (year)	Female proportion	Incidence type	Sample	POAG	PACG	Secondary glaucoma
**G20**	Vijaya L, et al.	2014	India	SEAR	LM-SDI	Both	2001-2004	6 years	Yes, all	Yes, all	Yes, all	Yes, all	Yes, all	40+	0.55	CI	4316	129	-	-
**G21**	Newman-Casey PA, et al.	2014	United states	AMR	H-SDI	Mixed	2001	6.4 year	No	No	No	No	No	50+	1	CI	152163	2925	-	-
**G22**	Kashiwagi K, et al.	2013	Japan	WPR	H-SDI	Urban	2005	5 years	Yes, suspect	Yes, suspect	Yes, all	Yes, suspect	Yes, all	40+	0.74	CI	331	-	4	-
**G23**	Vijaya L, et al.	2013	India	SEAR	LM-SDI	Both	2001-2004	6 years	Yes, all	Yes, all	Yes, all	Yes, suspect	Yes, all	40+	0.54	CI	3350	-	9	-

Article ID	Author	Publish year	Country	WHO region	SDI region	Urban/ Rural	Baseline	Follow-up time	ACA/ depth evaluation	IOP measurement	Optic disc evaluation	Visual field testing	Visual acuity	Age range (year)	Female proportion	Incidence type	Sample	POAG	PACG	Secondary glaucoma
**G24***	Marcus MW, et al.	2012	Netherlands	EUR	H-SDI	Urban	1991-1993	9.8 years	Yes, all	Yes, all	Yes, all	Yes, all	Yes, all	55+	0.584	CI	3939	108	-	-
**G25***	Marcus MW, et al.	2012	Netherlands	EUR	H-SDI	Urban	1991-1993	9.8 years	Yes, all	Yes, all	Yes, all	Yes, all	Yes, all	55+	0.584	CI	3939	108	-	-
**G26***	Marcus MW, et al.	2012	Netherlands	EUR	H-SDI	Urban	1991-1993	9.8 years	Yes, all	Yes, all	Yes, all	Yes, all	Yes, all	55+	0.584	CI	3939	108	-	-
**G27**	Ramdas WD, et al.	2012	Netherlands	EUR	H-SDI	Urban	1991-1993	9.7 years	Yes, all	Yes, all	Yes, all	Yes, all	Yes, all	55+	0.59	CI	3502	91	-	-
**G28**	Cedrone C, et al.	2012	Italy	EUR	HM-SDI	Urban	1988	12 years	No	Yes, all	Yes, all	Yes, suspect	No	40+	0.5879	CI	398	15	2	-
**G29**	Wise LA, et al.	2011	United states	AMR	HM-	Mixed	1995	NA	No	No	No	No	No	21-69	1	CI	32570	366	-	-

Article ID	Author	Publish year	Country	WHO region	SDI region	Urban/ Rural	Baseline	Follow-up time	ACA/ depth evaluation	IOP measurement	Optic disc evaluation	Visual field testing	Visual acuity	Age range (year)	Female proportion	Incidence type	Sample	POAG	PACG	Secondary glaucoma
					SDI															
**G29**	Wise LA, et al.	2011	United states	AMR	HM-SDI	Mixed	1995	NA	No	No	No	No	No	21-69	1	IR	416171	366	-	-
**G30**	Pasquale LR, et al.	2011	United states	AMR	HM-SDI	Mixed	1976	NA	Yes, all	Yes, all	Yes, all	Yes, all	No	40-55	1	CI	79440	813	-	-
**G31**	Yip JLY, et al.	2011	Mongolia	WPR	LM-SDI	Both	1999	3 years	Yes, all	Yes, all	Yes, all	Yes, suspect	Yes, all	51-101	0.6422	CI	1892	-	29	-
**G32**	Pasquale LR, et al.	2010	United states	AMR	HM-SDI	Mixed	1976	NA	Yes, all	Yes, all	Yes, all	Yes, all	No	40+	1	CI	78777	642	-	-

Article ID	Author	Publish year	Country	WHO region	SDI region	Urban/ Rural	Baseline	Follow-up time	ACA/ depth evaluation	IOP measurement	Optic disc evaluation	Visual field testing	Visual acuity	Age range (year)	Female proportion	Incidence type	Sample	POAG	PACG	Secondary glaucoma
**G32**	Pasquale LR, et al.	2010	United states	AMR	HM-SDI	Mixed	1986	NA	Yes, all	Yes, all	Yes, all	Yes, all	No	40+	0	CI	41352	338	-	-
**G32**	Pasquale LR, et al.	2010	United states	AMR	HM-SDI	Mixed	1976 (NHS), 1986 (HPFS)	NA	Yes, all	Yes, all	Yes, all	Yes, all	No	40+	0.656	IR	1610334	980	-	-
**G33**	Kang JH, et al.	2008	United states	AMR	HM-SDI	Mixed	1980	NA	Yes, all	Yes, all	Yes, all	Yes, all	No	40-55	1	CI	79120	658	-	-
**G33**	Kang JH, et al.	2008	United states	AMR	HM-SDI	Mixed	1986	NA	Yes, all	Yes, all	Yes, all	Yes, all	No	40-75	0	CI	42052	353	-	-

Article ID	Author	Publish year	Country	WHO region	SDI region	Urban/ Rural	Baseline	Follow-up time	ACA/ depth evaluation	IOP measurement	Optic disc evaluation	Visual field testing	Visual acuity	Age range (year)	Female proportion	Incidence type	Sample	POAG	PACG	Secondary glaucoma
**G33**	Kang JH, et al.	2008	United states	AMR	HM-SDI	Mixed	1980 (NHS), 1986 (HPFS)	NA	Yes, all	Yes, all	Yes, all	Yes, all	No	40-75	0.653	IR	1647311	1011	-	-
**G34**	Leske MC, et al.	2008	Barbados	AMR	M-SDI	Mixed	1987-1992	9 years	No	Yes, all	Yes, all	Yes, all	No	40-84	0.59	CI	3222	125	-	-
**G35**	Hitzl W, et al.	2007	Austria	EUR	HM-SDI	Mixed	December 1996-November 2000	5.3 years	Yes, all	Yes, all	Yes, all	Yes, all	Yes, all	40+	NA	CI	848	9	-	-
**G36**	Hennis A, et al.	2007	Barbados	AMR	M-SDI	Mixed	Apr 1988-May 1992	9 years	No	Yes, all	Yes, all	Yes, all	Yes, all	40-84	NA	CI	4314	125	-	-

Article ID	Author	Publish year	Country	WHO region	SDI region	Urban/ Rural	Baseline	Follow-up time	ACA/ depth evaluation	IOP measurement	Optic disc evaluation	Visual field testing	Visual acuity	Age range (year)	Female proportion	Incidence type	Sample	POAG	PACG	Secondary glaucoma
**G37**	Nemesure B, et al.	2007	Barbados	AMR	M-SDI	Mixed	1987-1992	9 years	No	Yes, all	Yes, all	Yes, all	Yes, all	40-84	0.59	CI	3222	125	-	-
**G38**	Leske MC, et al.	2007	Barbados	AMR	M-SDI	Mixed	1987-1992	9 years	No	Yes, all	Yes, all	Yes, all	Yes, all	40-84	0.59	CI	3222	125	-	-
**G39**	Muskens RPHM, et al.	2007	Netherlands	EUR	H-SDI	Urban	1990-1993	6.5 years	Yes, all	Yes, all	Yes, all	Yes, all	No	55+	0.59	CI	3842	87	-	-
**G40**	Papadopoulos M, et al.	2007	United Kingdom	EUR	HM-SDI	Mixed	2001	1 year	No	No	No	No	No	16-	NA	CI	1354574	-	-	52
**G41**	de Voogd S, et al.	2006	Netherlands	EUR	H-SDI	Urban	1990-1993	6.5 years	Yes, all	Yes, all	Yes, all	Yes, all	No	55+	0.58	CI	3842	87	-	-

Article ID	Author	Publish year	Country	WHO region	SDI region	Urban/Rural	Baseline	Follow-up time	ACA/depth evaluation	IOP measurement	Optic disc evaluation	Visual field testing	Visual acuity	Age range (year)	Female proportion	Incidence type	Sample	POAG	PACG	Secondary glaucoma
**G42**	de Voogd S, et al.	2006	Netherlands	EUR	H-SDI	Urban	1990-1993	6.5 years	Yes, all	Yes, all	Yes, all	Yes, all	No	55+	0.6	CI	3837	87	-	-
**G43**	Pasquale LR, et al.	2006	United states	AMR	HM-SDI	Mixed	1976	NA	Yes, all	Yes, all	Yes, all	Yes, all	No	40-55	1	CI	76318	429	-	-
**G43**	Pasquale LR, et al.	2006	United states	AMR	HM-SDI	Mixed	1976	NA	Yes, all	Yes, all	Yes, all	Yes, all	No	40-55	1	IR	998292	429	-	-
**G44**	de Voogd S, et al.	2005	Netherlands	EUR	H-SDI	Urban	1990-1993	6.5 years	Yes, all	Yes, all	Yes, all	Yes, all	Yes, all	55+	0.58	CI	3842	87	-	-
**G44**	de Voogd S, et al.	2005	Netherlands	EUR	H-SDI	Urban	1990-1993	6.5 years	Yes, all	Yes, all	Yes, all	Yes, all	Yes, all	55+	0.58	IR	24539	87	-	-

Article ID	Author	Publish year	Country	WHO region	SDI region	Urban/ Rural	Baseline	Follow-up time	ACA/ depth evaluation	IOP measurement	Optic disc evaluation	Visual field testing	Visual acuity	Age range (year)	Female proportion	Incidence type	Sample	POAG	PACG	Secondary glaucoma
**G45**	Ikram MK, et al.	2005	Netherlands	EUR	H-SDI	Urban	1990-1993	6.5 years	Yes, all	Yes, all	Yes, all	Yes, all	Yes, all	55+	0.58	CI	3469	74	-	-
**G46**	Sloan FA, et al.	2003	United states	AMR	HM-SDI	Mixed	1991	NA	No	No	No	No	No	NA	NA	CI	20019	1982	-	-
**G46**	Sloan FA, et al.	2003	United states	AMR	HM-SDI	Mixed	1992	NA	No	No	No	No	No	NA	NA	CI	17570	562	-	-
**G46**	Sloan FA, et al.	2003	United states	AMR	HM-SDI	Mixed	1993	NA	No	No	No	No	No	NA	NA	CI	16079	386	-	-
**G46**	Sloan FA, et al.	2003	United states	AMR	HM-SDI	Mixed	1994	NA	No	No	No	No	No	NA	NA	CI	14583	350	-	-

Article ID	Author	Publish year	Country	WHO region	SDI region	Urban/ Rural	Baseline	Follow-up time	ACA/ depth evaluation	IOP measurement	Optic disc evaluation	Visual field testing	Visual acuity	Age range (year)	Female proportion	Incidence type	Sample	POAG	PACG	Secondary glaucoma
**G46**	Sloan FA, et al.	2003	United states	AMR	HM-SDI	Mixed	1995	NA	No	No	No	No	No	NA	NA	CI	13094	380	-	-
**G46**	Sloan FA, et al.	2003	United states	AMR	HM-SDI	Mixed	1996	NA	No	No	No	No	No	NA	NA	CI	11593	232	-	-
**G46**	Sloan FA, et al.	2003	United states	AMR	HM-SDI	Mixed	1997	NA	No	No	No	No	No	NA	NA	CI	10240	164	-	-
**G46**	Sloan FA, et al.	2003	United states	AMR	HM-SDI	Mixed	1998	NA	No	No	No	No	No	NA	NA	CI	9013	162	-	-
**G46**	Sloan FA, et al.	2003	United states	AMR	HM-	Mixed	1998	NA	No	No	No	No	No	NA	NA	CI	7988	112	-	-

Article ID	Author	Publish year	Country	WHO region	SDI region	Urban/ Rural	Baseline	Follow-up time	ACA/ depth evaluation	IOP measurement	Optic disc evaluation	Visual field testing	Visual acuity	Age range (year)	Female proportion	Incidence type	Sample	POAG	PACG	Secondary glaucoma
**G47**	Nemesure B, et al.	2003	Barbados	AMR	SDI M-SDI	Mixed	1988-1992	NA	Yes, all	Yes, all	Yes, all	Yes, all	Yes, all	40-84	0.6	CI	2495	18	-	-
**G48**	Kang JH, et al.	2003	United states	AMR	H M-SDI	Mixed	1976	NA	Yes, all	Yes, all	Yes, all	Yes, all	No	40-55	1	CI	73560	286	-	-
**G48**	Kang JH, et al.	2003	United states	AMR	H M-SDI	Mixed	1986	NA	Yes, all	Yes, all	Yes, all	Yes, all	No	40-75	0	CI	37655	164	-	-
**G48**	Kang JH, et al.	2003	United states	AMR	H M-SDI	Mixed	1976 (NHS), 1986 (HPFS)	NA	Yes, all	Yes, all	Yes, all	Yes, all	No	40-75	0.6614	IR	1035227	450	-	-

Article ID	Author	Publish year	Country	WHO region	SDI region	Urban/ Rural	Baseline	Follow-up time	ACA/ depth evaluation	IOP measurement	Optic disc evaluation	Visual field testing	Visual acuity	Age range (year)	Female proportion	Incidence type	Sample	POAG	PACG	Secondary glaucoma
**G49**	Leske MC, et al.	2002	Barbados	AMR	M-SDI	Mixed	1988-1992	4 years	Yes, all	Yes, all	Yes, all	Yes, all	Yes, all	40-84	NA	CI	2989	67	-	-
**G50**	Leske MC, et al.	2001	Barbados	AMR	M-SDI	Mixed	1988-1992	4 years	Yes, all	Yes, all	Yes, all	Yes, all	Yes, all	40-84	0.5905	CI	2989	67	-	-

Notes: WHO, World Health Organization; EUR, European Region; AMR, Region of the Americas; SEAR, South-East Asian Region; WPR, Western Pacific Region; SDI, social demographic index; H-SDI, high SDI; HM-SDI, high-middle SDI; M-SDI, middle SDI; LM-SDI, low-middle SDI; ACA, Anterior chamber angle; IOP, intraocular pressure; POAG, primary open-angle glaucoma; PACG, primary angle-closure glaucoma; CI, cumulative incidence; IR, incidence rate. *Article G24, G25 and G26 had different inclusion and exclusion criteria.

Table S7. Main characteristics of the included articles

Characteristics	Articles reported glaucoma incidence (n=50)	Articles reported associated factors (n=38)
	N (%)	N (%)
**Publish year**		
1990-1999	0 (0.0%)	0 (0.0%)
2000-2009	18 (36.0%)	13 (34.2%)
2010-2022	32 (64.0%)	25 (65.8%)
**WHO region**		
EUR	16 (32.0%)	12 (31.6%)
AFR	0 (0.0%)	0 (0.0%)
EMR	0 (0.0%)	0 (0.0%)
AMR	22 (44.0%)	19 (50.0%)
SEAR	3 (6.0%)	2 (5.3%)
WPR	9 (18.0%)	5 (13.2%)
**SDI region**		
H-SDI	18 (36.0%)	16 (42.1%)
HM-SDI	16 (32.0%)	9 (23.7%)
M-SDI	12 (24.0%)	10 (26.3%)
LM-SDI	5 (10.0%)	3 (7.9%)
**Study setting**		
Urban	12 (24.0%)	10 (26.3%)
Rural	4 (8.0%)	3 (7.9%)
Mixed	34 (68.0%)	25 (65.8%)

Characteristics	Articles reported glaucoma incidence (n=50)	Articles reported associated factors (n=38)
	N (%)	N (%)
**Subtype**		
POAG	43 (86.0%)	37 (97.4%)
PACG	10 (20.0%)	3 (7.9%)
Secondary glaucoma	1 (2.0%)	0 (0.0%)
**Anterior chamber angle/depth evaluation**		
Yes, all	36 (72.0%)	29 (76.3%)
Yes, suspects	2 (4.0%)	0 (0.0%)
No	12 (24.0%)	9 (23.7%)
**IOP measurement**		
Yes, all	41 (82.0%)	32 (84.2%)
Yes, suspects	2 (4.0%)	1 (2.6%)
No	7 (14.0%)	5 (13.2%)
**Optic disc evaluation**		
Yes, all	44 (88.0%)	34 (89.5%)
Yes, suspects	0 (0.0%)	0 (0.0%)
No	6 (12.0%)	4 (10.5%)
**Visual field testing**		
Yes, all	36 (72.0%)	29 (76.3%)
Yes, suspects	8 (16.0%)	5 (13.2%)
No	6 (12.0%)	4 (10.5%)
**Visual acuity**		
Yes, all	24 (48.0%)	16 (42.1%)

Characteristics	Articles reported glaucoma incidence (n=50)	Articles reported associated factors (n=38)
	N (%)	N (%)
Yes, suspects	0 (0.0%)	0 (0.0%)
No	26 (52.0%)	22 (57.9%)
**Quality score**		
9	22 (44.0%)	17 (44.7%)
8	19 (38.0%)	16 (43.2%)
7	7 (14.0%)	5 (13.5%)
6	1 (2.0%)	0 (0.0%)
5	1 (2.0%)	0 (0.0%)

Notes: WHO, World Health Organization; EUR, European Region; AFR, African Region; EMR, Eastern Mediterranean Region; AMR, Region of the Americas; SEAR, South-East Asian Region; WPR, Western Pacific Region; SDI, social demographic index; H-SDI, high SDI; HM-SDI, high-middle SDI; M-SDI, middle SDI; LM-SDI, low-middle SDI; POAG, primary open-angle glaucoma; PACG, primary angle-closure glaucoma; IOP, intraocular pressure. Four articles provided incidence data for both POAG and PACG.

Table S8. Quality assessment of included articles (n=50)

Article ID	Author	Year Published	Selection				Comparability	Outcome			NOS score
			S1	S2	S3	S4	C1	O1	O2	O3	
**G1**	Zhang Y, et al.	2022	*	*	*	*	**	*	*	*	9
**G2**	Hanyuda A, et al.	2022		*	*	*	**	*	*	*	8
**G3**	Teo ZL, et al.	2022	*	*	*	*	**	*	*	*	9
**G4**	Azizova TV, et al.	2022		*	*	*	**	*	*	*	8
**G5**	Choudhari NS, et al.	2021	*	*	*	*	**	*	*	*	9
**G6**	Bragin EV, et al.	2021		*	*	*		*	*	*	6
**G7**	Koh V, et al.	2021	*	*	*	*	**	*	*	*	9
**G8**	Founti P, et al.	2021	*	*	*	*	**	*	*		8
**G9**	Hanyuda A, et al.	2020		*	*	*	**	*	*	*	8
**G10**	Jung Y, et al.	2020	*	*	*	*	**	*	*	*	9
**G11**	Bragin EV, et al.	2019		*	*	*	**	*	*	*	8
**G12**	Wang W, et al.	2018	*	*	*	*	**	*	*	*	9
**G13**	Kang JH, et al.	2018		*	*	*	**	*	*	*	8
**G14**	Lee NY, et al.	2017	*	*	*	*	**	*	*	*	9
**G15**	Pan CW, et al.	2017	*	*	*	*	**	*	*	*	9
**G16**	Kang JH, et al.	2016		*	*	*	**	*	*	*	8
**G17**	Li L, et al.	2016	*	*	*	*	**	*	*	*	9
**G18**	Pasquale LR, et al.	2016		*		*	**	*	*	*	7
**G19**	Kang JH, et al.	2015		*		*	**	*	*	*	7
**G20**	Vijaya L, et al.	2014	*	*	*	*	**	*	*	*	9

Article ID	Author	Year Published	Selection				Comparability	Outcome			NOS score
			S1	S2	S3	S4	C1	O1	O2	O3	
**G21**	Newman-Casey PA, et al.	2014	*	*	*	*	**	*	*	*	9
**G22**	Kashiwagi K, et al.	2013	*	*	*	*	*	*	*		7
**G23**	Vijaya L, et al.	2013	*	*	*	*	**	*	*	*	9
**G24**	Marcus MW, et al.	2012	*	*	*	*	**	*	*		8
**G25**	Marcus MW, et al.	2012	*	*	*	*	**	*	*		8
**G26**	Marcus MW, et al.	2012	*	*	*	*	**	*	*		8
**G27**	Ramdas WD, et al.	2012	*	*	*	*	**	*	*		8
**G28**	Cedrone C, et al.	2012	*	*	*	*	**	*	*	*	9
**G29**	Wise LA, et al.	2011	*	*		*	**	*	*	*	8
**G30**	Pasquale LR, et al.	2011		*		*	**	*	*	*	7
**G31**	Yip JLY, et al.	2011	*	*	*	*	**	*	*	*	9
**G32**	Pasquale LR, et al.	2010		*	*	*	**	*	*	*	8
**G33**	Kang JH, et al.	2008		*		*	**	*	*	*	7
**G34**	Leske MC, et al.	2008	*	*	*	*	**	*	*	*	9
**G35**	Hitzl W, et al.	2007	*	*	*	*		*	*	*	7
**G36**	Hennis A, et al.	2007	*	*		*	**	*	*	*	8
**G37**	Nemesure B, et al.	2007	*	*	*	*	**	*	*	*	9
**G38**	Leske MC, et al.	2007	*	*	*	*	**	*	*	*	9
**G39**	Muskens RPHM, et al.	2007	*	*	*	*	**	*	*	*	9
**G40**	Papadopoulos M, et al.	2007	*	*	*	*	**	*		*	8
**G41**	de Voogd S, et al.	2006	*	*	*	*	**	*	*	*	9
**G42**	de Voogd S, et al.	2006	*	*	*	*	**	*	*		8
**G43**	Pasquale LR, et al.	2006		*	*	*	**	*	*	*	8
**G44**	de Voogd S, et al.	2005	*	*	*	*	**	*	*		8

Article ID	Author	Year Published	Selection				Comparability	Outcome			NOS score
			S1	S2	S3	S4	C1	O1	O2	O3	
**G45**	Ikram MK, et al.	2005	*	*	*	*	**	*	*		8
**G46**	Sloan FA, et al.	2003	*	*		*		*	*		5
**G47**	Nemesure B, et al.	2003	*	*	*	*	**	*	*	*	9
**G48**	Kang JH, et al.	2003		*		*	**	*	*	*	7
**G49**	Leske MC, et al.	2002	*	*	*	*	**	*	*	*	9
**G50**	Leske MC, et al.	2001	*	*	*	*	**	*	*	*	9

Notes: S1: Representativeness of the exposed cohort; S2: Selection of the non exposed cohort; S3: Ascertainment of exposure; S4: Demonstration that outcome of interest was not present at start of study; C1: Comparability of cohorts on the basis of the design or analysis; O1: Assessment of outcome; O2: Was follow-up long enough for outcomes to occur (3 year); O3: Adequacy of follow up of cohorts (70% or no description).

Table S9. Estimated incidence rate of primary open-angle glaucoma in adults aged 40–79 years in 2019, by WHO region (per 10,000 person-years)

Sex	Age group	AFR	AMR	SEAR	EUR	EMR	WPR
Male	40-44 years	8.36 (1.66-18.48)	3.75 (0.95-7.61)	5.80 (1.52-11.65)	1.50 (0.10-4.85)	5.98 (1.14-13.49)	3.15 (0.83-6.61)
	45-49 years	15.84 (6.19-28.99)	8.83 (4.59-14.08)	12.04 (5.89-19.81)	5.01 (1.53-10.15)	12.17 (4.85-22.16)	7.92 (4.12-12.79)
	50-54 years	24.69 (12.29-40.75)	15.61 (10.00-22.28)	19.87 (11.86-29.57)	10.19 (4.84-17.14)	20.15 (10.46-32.60)	14.47 (9.28-20.70)
	55-59 years	35.07 (20.04-53.76)	24.01 (17.07-32.01)	29.35 (19.52-40.88)	17.18 (10.14-25.79)	29.93 (17.88-44.82)	22.48 (15.93-30.10)
	60-64 years	47.08 (29.35-68.54)	34.04 (25.69-43.49)	40.42 (28.73-53.84)	25.80 (17.09-36.09)	41.33 (27.01-58.51)	32.00 (24.03-41.10)
	65-69 years	60.96 (40.21-85.62)	45.86 (35.86-57.01)	53.47 (39.54-69.22)	36.06 (25.42-48.34)	54.56 (37.82-74.25)	43.52 (33.89-54.32)
	70-74 years	77.21 (53.57-104.76)	59.59 (48.24-72.10)	68.46 (52.69-86.05)	47.89 (35.36-62.10)	70.76 (51.10-93.51)	56.00 (44.71-68.56)
	75-79 years	94.46 (67.97-124.92)	74.83 (61.90-88.96)	84.78 (67.02-104.42)	61.27 (46.79-77.49)	87.8 (65.33-113.49)	70.04 (56.97-84.47)
	**40-79 years**	**29.16 (16.42-45.51)**	**23.62 (17.20-31.11)**	**26.94 (18.11-37.45)**	**20.28 (13.40-28.84)**	**25.76 (15.52-38.79)**	**23.35 (17.06-30.77)**
Female	40-44 years	14.94 (4.22-31.41)	5.56 (1.85-12.09)	10.79 (3.93-20.48)	3.61 (0.61-9.44)	11.60 (3.33-24.29)	6.41 (2.45-12.33)
	45-49 years	20.91 (7.98-39.53)	9.40 (3.97-17.35)	16.03 (7.60-27.22)	7.06 (2.25-14.37)	16.99 (6.77-31.58)	10.77 (5.44-17.88)
	50-54 years	27.82 (12.69-48.61)	14.11 (7.15-23.52)	22.21 (12.21-34.93)	11.39 (4.99-20.23)	23.43 (11.27-39.93)	16.14 (9.53-24.44)
	55-59 years	35.59 (18.30-58.52)	19.50 (11.03-30.46)	29.36 (17.75-43.69)	16.84 (8.94-27.14)	30.99 (16.77-49.61)	22.23 (14.33-31.85)
	60-64 years	44.39 (24.83-69.64)	25.72 (15.67-38.35)	37.46 (24.19-53.49)	23.23 (13.84-35.00)	39.14 (23.13-59.51)	29.18 (19.92-40.21)
	65-69 years	54.06 (32.22-81.63)	33.01 (21.32-47.33)	46.63 (31.56-64.54)	30.43 (19.48-43.78)	47.94 (30.26-69.88)	37.22 (26.57-49.69)
	70-74 years	65.03 (40.56-95.39)	41.00 (27.58-57.15)	56.51 (39.68-76.23)	38.25 (25.57-53.46)	58.50 (38.42-83.05)	45.30 (33.01-59.59)
	75-79 years	76.41 (49.62-109.14)	50.40 (35.21-68.39)	67.42 (48.71-89.10)	47.18 (32.73-64.26)	69.92 (47.44-97.03)	54.41 (40.37-70.62)
	**40-79 years**	**31.49 (15.78-52.92)**	**20.60 (12.29-31.53)**	**27.39 (16.54-41.03)**	**19.54 (11.49-30.09)**	**27.47 (14.71-44.62)**	**22.69 (14.93-32.26)**
Overall	40-44 years	9.88 (2.50-20.59)	4.05 (1.27-8.30)	6.98 (2.29-13.18)	2.12 (0.29-5.83)	7.32 (1.84-15.39)	3.99 (1.37-7.72)
	45-49 years	16.81 (6.84-30.25)	8.32 (4.06-13.93)	12.81 (6.49-20.73)	5.50 (1.82-10.80)	13.23 (5.55-23.57)	8.52 (4.59-13.50)
	50-54 years	24.86 (12.39-40.93)	13.79 (8.15-20.79)	19.92 (11.95-29.55)	10.21 (4.88-17.12)	20.58 (10.76-33.15)	14.49 (9.33-20.69)
	55-59 years	34.15 (19.33-52.57)	20.29 (13.33-28.64)	28.42 (18.83-39.68)	16.45 (9.62-24.80)	29.47 (17.50-44.23)	21.65 (15.29-29.08)

Sex	Age group	AFR	AMR	SEAR	EUR	EMR	WPR
	60-64 years	44.75 (27.51-65.68)	27.83 (19.45-37.65)	38.24 (26.97-51.19)	23.97 (15.67-33.78)	39.52 (25.58-56.27)	30.05 (22.40-38.80)
	65-69 years	56.77 (36.83-80.57)	36.69 (26.69-48.23)	49.67 (36.38-64.76)	32.70 (22.73-44.27)	50.84 (34.83-69.75)	40.03 (30.90-50.33)
	70-74 years	70.73 (48.17-97.17)	46.67 (35.23-59.67)	62.43 (47.54-79.13)	42.68 (31.04-55.95)	64.72 (46.19-86.26)	50.60 (39.98-62.49)
	75-79 years	85.08 (60.07-114.03)	58.00 (45.01-72.62)	76.18 (59.50-94.71)	53.65 (40.33-68.68)	79.33 (58.3-103.48)	62.20 (49.95-75.83)
	**40-79 years**	**29.08 (16.20-45.53)**	**21.03 (14.32-29.19)**	**26.27 (17.51-36.67)**	**19.51 (12.69-27.96)**	**25.62 (15.26-38.72)**	**22.45 (16.24-29.76)**

Notes: WHO, World Health Organization; EUR, European Region; AFR, African Region; EMR, Eastern Mediterranean Region; AMR, Region of the Americas; SEAR, South-East Asian Region; WPR, Western Pacific Region.

Table S10. Estimated incidence rate of primary open-angle glaucoma in adults aged 40–79 years in 2019, by SDI region (per 10,000 person-years)

Sex	Age group	High SDI	High-middle SDI	Middle SDI	Low-middle SDI	Low SDI
Male	40-44 years	0.52 (0.00-4.22)	1.74 (0.04-4.85)	3.50 (0.95-6.84)	6.32 (1.67-12.67)	11.35 (1.87-26.29)
	45-49 years	3.23 (0.02-9.27)	5.58 (2.05-10.21)	8.52 (4.69-13.14)	12.89 (6.22-21.33)	20.24 (6.64-39.47)
	50-54 years	7.80 (2.02-16.04)	11.20 (6.19-17.3)	15.25 (10.20-21.15)	21.04 (12.36-31.57)	30.42 (12.93-53.77)
	55-59 years	14.10 (6.12-24.44)	18.53 (12.12-26.01)	23.65 (17.39-30.77)	30.80 (20.16-43.30)	42.11 (20.89-69.25)
	60-64 years	22.04 (11.93-34.51)	27.50 (19.67-36.42)	33.67 (26.16-42.08)	42.15 (29.51-56.68)	55.32 (30.41-86.25)
	65-69 years	31.78 (19.39-46.61)	38.28 (28.81-48.89)	45.54 (36.48-55.54)	55.37 (40.44-72.29)	70.43 (41.52-105.64)
	70-74 years	43.46 (28.91-60.36)	51.03 (40.16-63.05)	59.41 (49.12-70.67)	70.65 (53.76-89.51)	87.68 (55.07-126.45)
	75-79 years	56.57 (39.86-75.64)	65.18 (52.78-78.76)	74.63 (62.91-87.35)	87.19 (68.22-108.16)	106.05 (69.78-148.51)
	**40-79 years**	**18.87 (10.87-29.43)**	**20.05 (14.07-27.18)**	**23.38 (17.54-30.07)**	**27.76 (18.31-39.03)**	**36.04 (17.85-60.04)**
Female	40-44 years	1.39 (0.00-6.07)	3.10 (0.52-6.83)	5.38 (2.26-9.20)	8.91 (3.37-15.99)	15.02 (3.69-31.50)
	45-49 years	4.01 (0.18-10.49)	6.61 (2.76-11.5)	9.83 (5.79-14.63)	14.57 (7.52-23.32)	22.48 (7.99-42.44)
	50-54 years	7.76 (1.96-15.98)	11.21 (6.22-17.23)	15.31 (10.34-21.10)	21.20 (12.57-31.60)	30.75 (13.16-54.02)
	55-59 years	12.62 (5.06-22.49)	16.91 (10.85-24.00)	21.90 (16.02-28.61)	28.90 (18.75-40.84)	40.06 (19.47-66.35)
	60-64 years	18.54 (9.28-30.08)	23.66 (16.50-31.88)	29.52 (22.68-37.25)	37.63 (25.90-51.18)	50.33 (26.76-79.70)
	65-69 years	25.58 (14.49-39.03)	31.55 (23.07-41.15)	38.30 (30.20-47.32)	47.51 (33.92-63.03)	61.77 (34.94-94.68)
	70-74 years	33.86 (21.05-48.99)	40.72 (31.12-51.46)	48.38 (39.29-58.44)	58.75 (43.59-75.84)	74.63 (44.81-110.45)
	75-79 years	43.06 (28.47-60.00)	50.77 (39.87-62.84)	59.30 (48.99-70.64)	70.76 (53.84-89.67)	88.15 (55.29-127.12)
	**40-79 years**	**16.35 (8.60-26.62)**	**19.33 (13.25-26.50)**	**21.75 (16.08-28.25)**	**26.49 (17.15-37.57)**	**35.29 (16.92-59.26)**
Overall	40-44 years	1.03 (0.00-5.32)	2.39 (0.27-5.80)	4.41 (1.59-7.99)	7.56 (2.49-14.27)	13.16 (2.77-28.87)
	45-49 years	3.69 (0.11-9.99)	6.08 (2.39-10.83)	9.15 (5.23-13.86)	13.70 (6.85-22.29)	21.35 (7.31-40.94)
	50-54 years	7.78 (1.98-16.00)	11.21 (6.21-17.26)	15.28 (10.27-21.13)	21.11 (12.46-31.58)	30.59 (13.04-53.90)
	55-59 years	13.21 (5.48-23.27)	17.71 (11.48-25.00)	22.80 (16.73-29.72)	29.89 (19.48-42.12)	41.08 (20.18-67.81)

Sex	Age group	High SDI	High-middle SDI	Middle SDI	Low-middle SDI	Low SDI
	60-64 years	19.90 (10.31-31.80)	25.49 (18.01-34.04)	31.64 (24.45-39.71)	39.96 (27.76-54.02)	52.81 (28.58-82.96)
	65-69 years	27.95 (16.37-41.93)	34.61 (25.68-44.66)	41.94 (33.36-51.46)	51.53 (37.25-67.77)	66.03 (38.17-100.07)
	70-74 years	37.57 (24.09-53.39)	45.28 (35.11-56.58)	53.78 (44.10-64.42)	64.68 (48.66-82.66)	80.95 (49.78-118.20)
	75-79 years	48.14 (32.75-65.88)	56.71 (45.19-69.41)	66.53 (55.56-78.53)	78.69 (60.78-98.60)	96.74 (62.24-137.38)
	**40-79 years**	**17.35 (9.50-27.74)**	**19.68 (13.65-26.83)**	**22.58 (16.82-29.18)**	**27.14 (17.75-38.32)**	**35.67 (17.39-59.65)**

Notes: SDI, social demographic index.

Table S11. Meta-analyses of risk factors for primary open-angle glaucoma

Risk factor	Article ID	Author	Year Published	Country	Meta-analysis
**Age - per year increase**					
	G7	Koh V, et al.	2021	Singapore	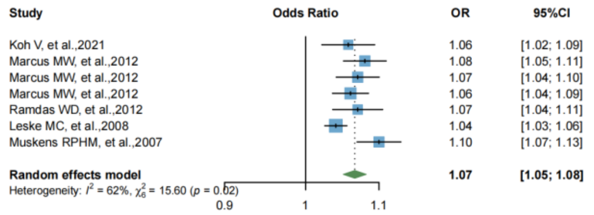
	G24	Marcus MW, et al.	2012	Netherlands	
	G25	Marcus MW, et al.	2012	Netherlands	
	G26	Marcus MW, et al.	2012	Netherlands	
	G27	Ramdas WD, et al.	2012	Netherlands	
	G34	Leske MC, et al.	2008	Barbados	
	G39	Muskens RPHM, et al.	2007	Netherlands	
**Age - 50-59 years vs 40-49 years**					
	G20	Vijaya L, et al.	2014	India	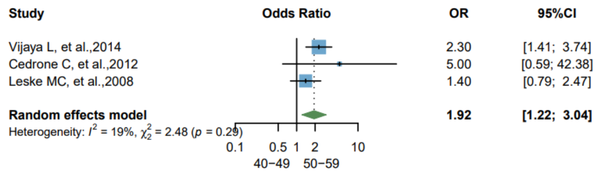
	G28	Cedrone C, et al.	2012	Italy	
	G34	Leske MC, et al.	2008	Barbados	
**Age - 60-69 years vs 40-49 years**					
	G20	Vijaya L, et al.	2014	India	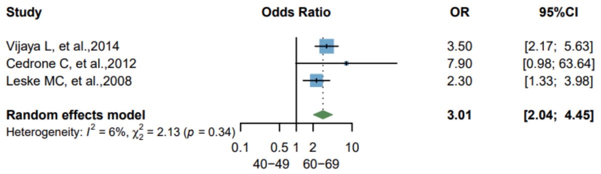
	G28	Cedrone C, et al.	2012	Italy	
	G34	Leske MC, et al.	2008	Barbados	
**Age - 70+ years vs 40-49 years**					
	G20	Vijaya L, et al.	2014	India	

Risk factor	Article ID	Author	Year Published	Country	Meta-analysis
	G28	Cedrone C, et al.	2012	Italy	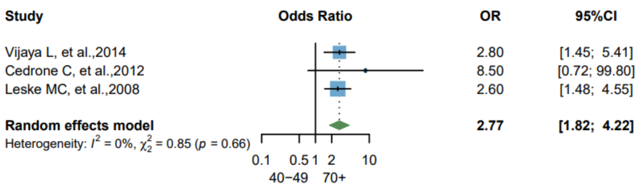
	G34	Leske MC, et al.	2008	Barbados	
**Female**					
	G11	Bragin EV, et al.	2019	Russia	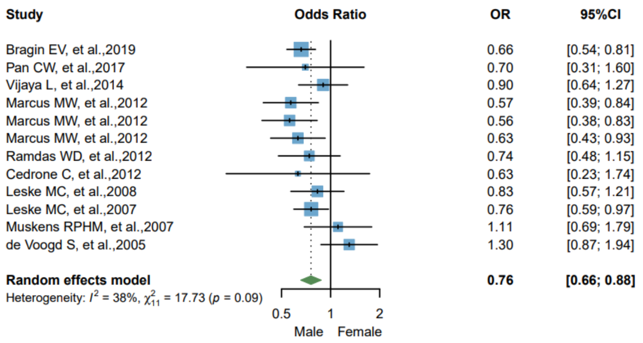
	G15	Pan CW, et al.	2017	China	
	G20	Vijaya L, et al.	2014	India	
	G24	Marcus MW, et al.	2012	Netherlands	
	G25	Marcus MW, et al.	2012	Netherlands	
	G26	Marcus MW, et al.	2012	Netherlands	
	G27	Ramdas WD, et al.	2012	Netherlands	
	G28	Cedrone C, et al.	2012	Italy	
	G34	Leske MC, et al.	2008	Barbados	
	G38	Leske MC, et al.	2007	Barbados	
	G39	Muskens RPHM, et al.	2007	Netherlands	
	G44	de Voogd S, et al.	2005	Netherlands	
**IOP (per 1 mmHg increase)**					
	G7	Koh V, et al.	2021	Singapore	
	G24	Marcus MW, et al.	2012	Netherlands	
	G25	Marcus MW, et al.	2012	Netherlands	
	G27	Ramdas WD, et al.	2012	Netherlands	

Risk factor	Article ID	Author	Year Published	Country	Meta-analysis
	G34	Leske MC, et al.	2008	Barbados	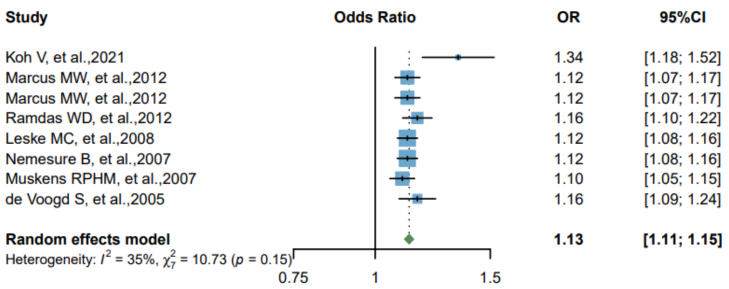
	G37	Nemesure B, et al.	2007	Barbados	
	G39	Muskens RPHM, et al.	2007	Netherlands	
	G44	de Voogd S, et al.	2005	Netherlands	
**IOP treatment**					
	G24	Marcus MW, et al.	2012	Netherlands	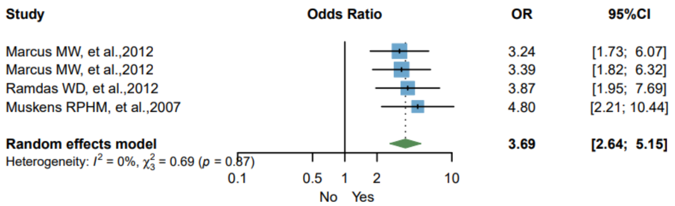
	G25	Marcus MW, et al.	2012	Netherlands	
	G27	Ramdas WD, et al.	2012	Netherlands	
	G39	Muskens RPHM, et al.	2007	Netherlands	
**Famliy history of glaucoma**					
	G19	Kang JH, et al.	2015	United states	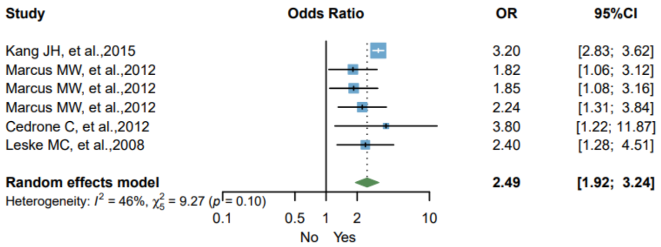
	G24	Marcus MW, et al.	2012	Netherlands	
	G25	Marcus MW, et al.	2012	Netherlands	
	G26	Marcus MW, et al.	2012	Netherlands	
	G28	Cedrone C, et al.	2012	Italy	
	G34	Leske MC, et al.	2008	Barbados	
**Myopia**					

Risk factor	Article ID	Author	Year Published	Country	Meta-analysis
	G15	Pan CW, et al.	2017	China	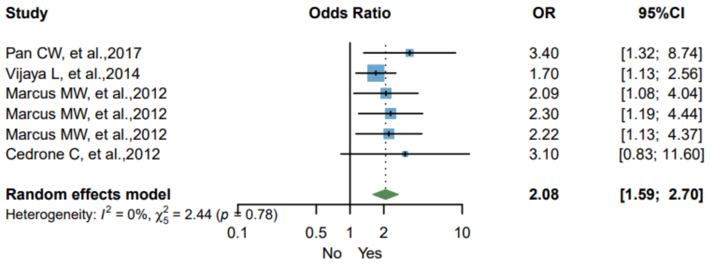
	G20	Vijaya L, et al.	2014	India	
	G24	Marcus MW, et al.	2012	Netherlands	
	G25	Marcus MW, et al.	2012	Netherlands	
	G26	Marcus MW, et al.	2012	Netherlands	
	G28	Cedrone C, et al.	2012	Italy	
**Diabetes**					
	G15	Pan CW, et al.	2017	China	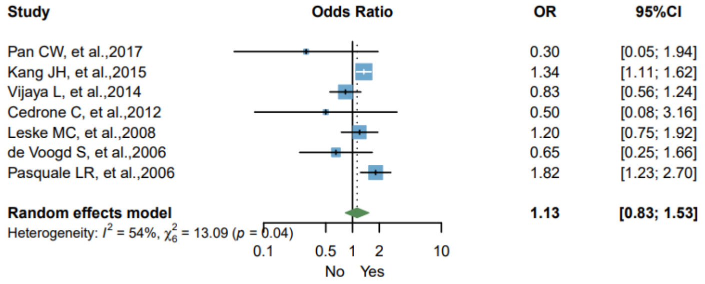
	G19	Kang JH, et al.	2015	United states	
	G20	Vijaya L, et al.	2014	India	
	G28	Cedrone C, et al.	2012	Italy	
	G34	Leske MC, et al.	2008	Barbados	
	G42	de Voogd S, et al.	2006	Netherlands	
	G43	Pasquale LR, et al.	2006	United states	
**Hypertension**					
	G15	Pan CW, et al.	2017	China	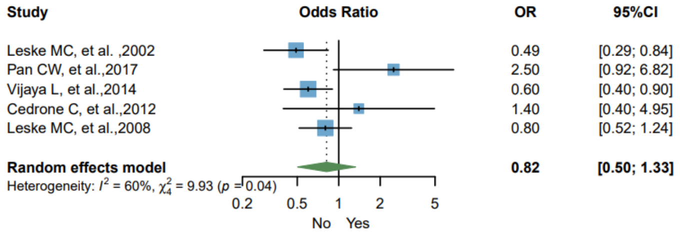
	G20	Vijaya L, et al.	2014	India	
	G28	Cedrone C, et al.	2012	Italy	
	G34	Leske MC, et al.	2008	Barbados	
	G49	Leske MC, et al.	2002	Barbados	

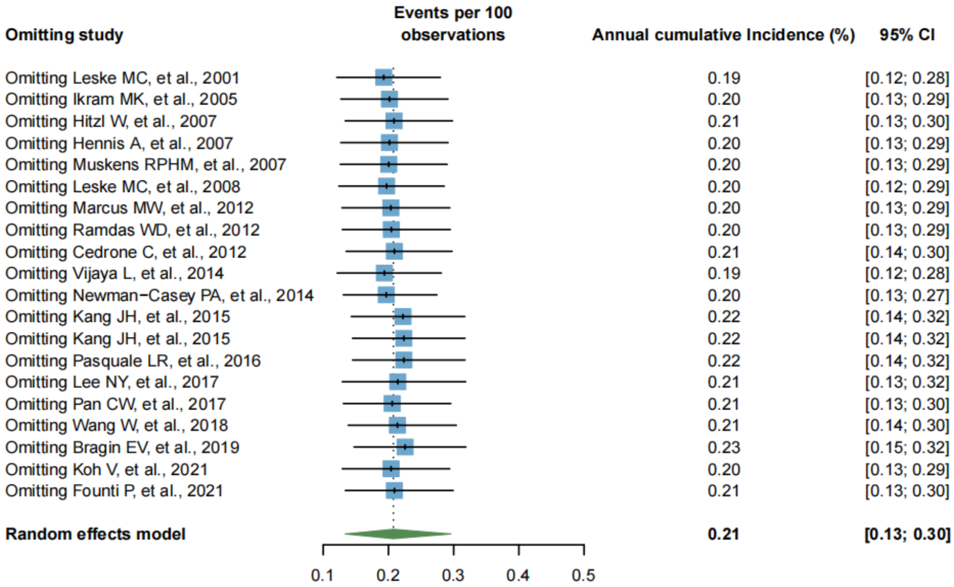

Figure S1. Leave-one-out sensitivity analysis for the pooled annual cumulative incidence of primary open-angle glaucoma.

(A)

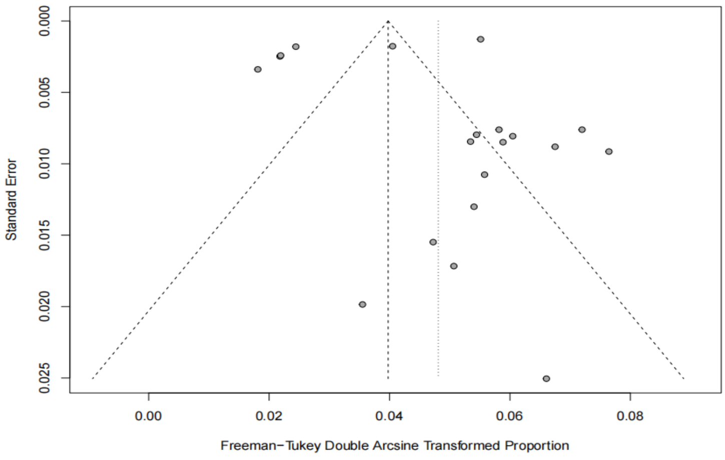

(B)

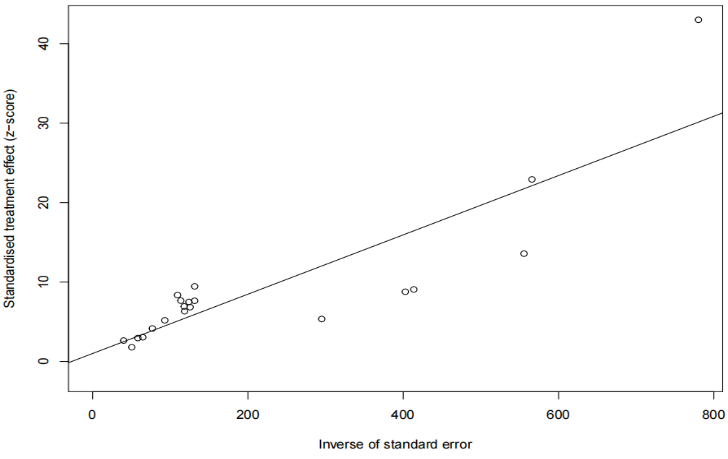

(C)

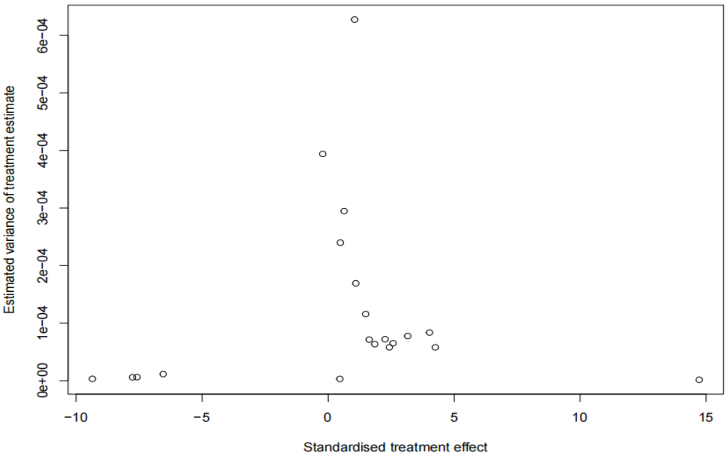

Figure S2. Publication bias test for the pooled annual cumulative incidence of primary open-angle glaucoma.

Notes: (A) Funnel plot; (B) Egger’s regression test; (C) Begg's rank correlation test.

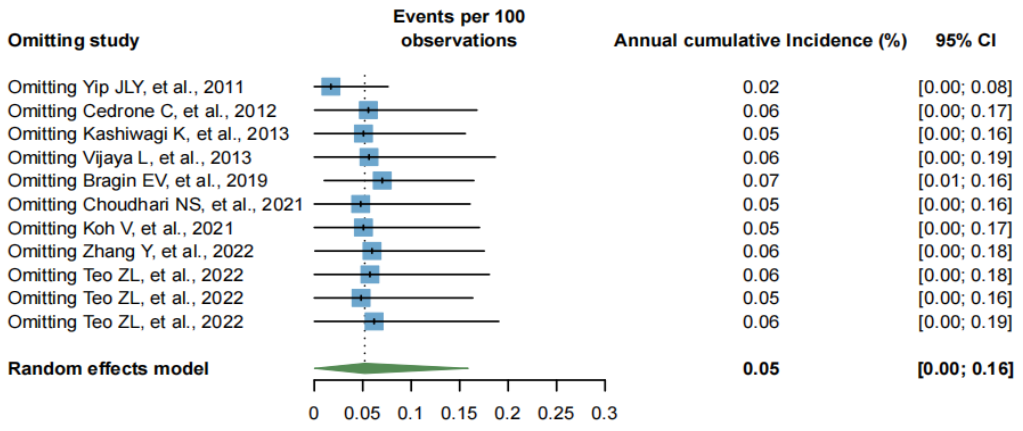

Figure S3. Leave-one-out sensitivity analysis for the pooled annual cumulative incidence of primary angle-closure glaucoma.

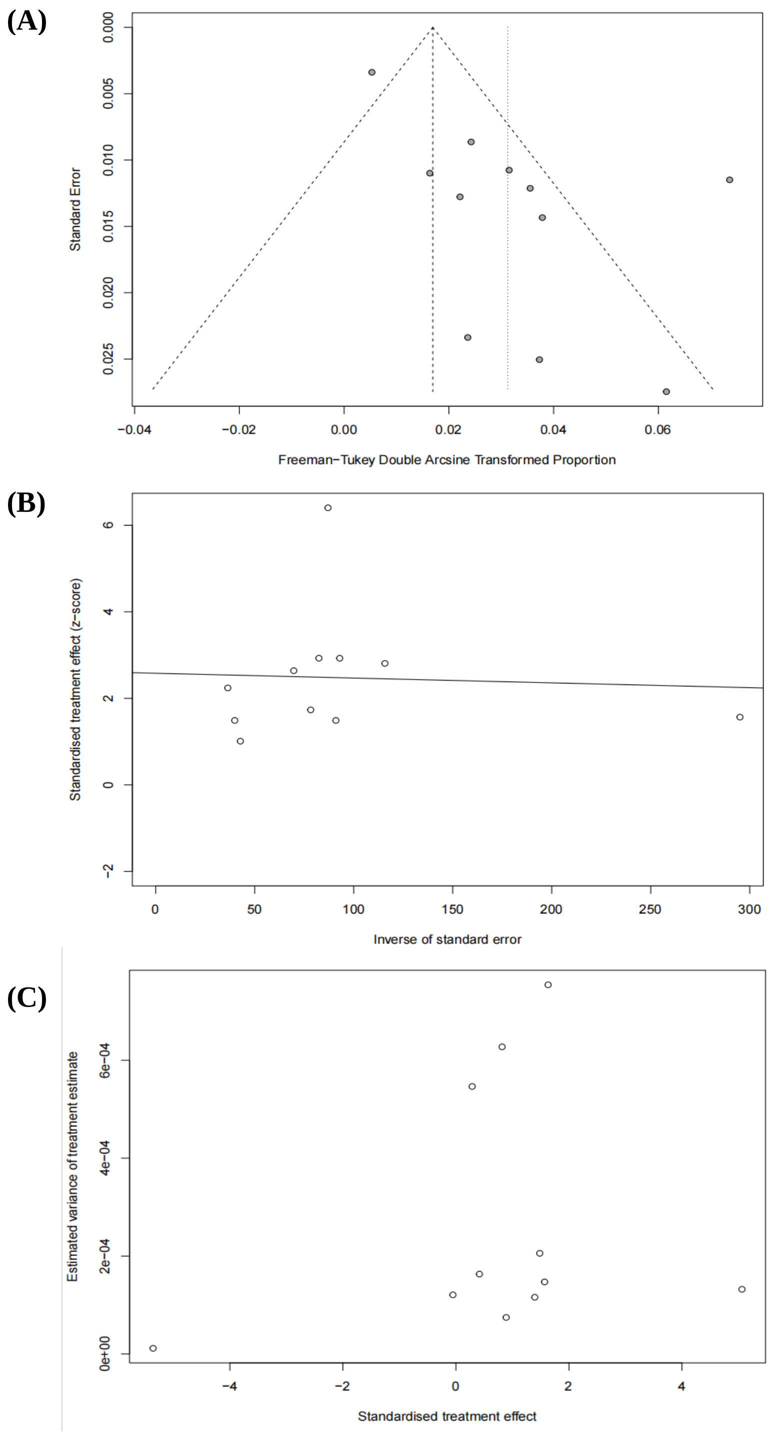

Figure S4. Publication bias test for the pooled annual cumulative incidence of primary angle-closure glaucoma.

Notes: (A) Funnel plot; (B) Egger’s regression test; (C) Begg's rank correlation test.
